# Multilevel visual motion opponency in *Drosophila*

**DOI:** 10.1038/s41593-023-01443-z

**Published:** 2023-10-02

**Authors:** Georg Ammer, Etienne Serbe-Kamp, Alex S. Mauss, Florian G. Richter, Sandra Fendl, Alexander Borst

**Affiliations:** 1 Max Planck Institute for Biological Intelligence, Martinsried, Germany; 2 Ludwig Maximilian University of Munich, Munich, Germany

**Keywords:** Neural circuits, Inhibition-excitation balance, Motion detection, Sensory processing

## Abstract

Inhibitory interactions between opponent neuronal pathways constitute a common circuit motif across brain areas and species. However, in most cases, synaptic wiring and biophysical, cellular and network mechanisms generating opponency are unknown. Here, we combine optogenetics, voltage and calcium imaging, connectomics, electrophysiology and modeling to reveal multilevel opponent inhibition in the fly visual system. We uncover a circuit architecture in which a single cell type implements direction-selective, motion-opponent inhibition at all three network levels. This inhibition, mediated by GluClα receptors, is balanced with excitation in strength, despite tenfold fewer synapses. The different opponent network levels constitute a nested, hierarchical structure operating at increasing spatiotemporal scales. Electrophysiology and modeling suggest that distributing this computation over consecutive network levels counteracts a reduction in gain, which would result from integrating large opposing conductances at a single instance. We propose that this neural architecture provides resilience to noise while enabling high selectivity for relevant sensory information.

## Abstract (web summary)

A single cell type in the *Drosophila* visual system implements motion-opponent inhibition at multiple consecutive circuit levels. This neural architecture enables high stimulus selectivity without compromising sensitivity under noisy conditions.

## Main

The nervous system can be viewed as being modularly organized into neural circuit motifs—repeated network structures with similar connectivity patterns—that perform canonical neural computations^1–4^. Understanding the structure and function of such circuit motifs might provide a conceptual link between the function of individual neurons and the entire brain. Inhibitory interactions between oppositely tuned channels are one classical example of a common circuit motif. In color vision, for example, opponent inhibition between channels tuned to different spectral wavelengths allows the circuit to disambiguate changes in spectral information from changes in relative brightness^5–7^. In the barn owl, a midbrain circuit involved in attention features a ‘reciprocal inhibition of inhibition’ connectivity motif that allows stimulus segregation into ‘strongest’ and ‘others’^8^. A similar network motif in the zebrafish hindbrain ensures that the animal escapes to the correct direction in response to a threatening stimulus^9^. Opponent inhibition also occurs between choice-selective neurons in the mouse posterior parietal cortex where it leads to amplified separation of oppositely tuned neural populations^10^. However, the aforementioned circuits are difficult to manipulate experimentally; in most cases, their synaptic architectures and the cellular and biophysical mechanisms that give rise to opponency are unknown.

**Fig. 1:**
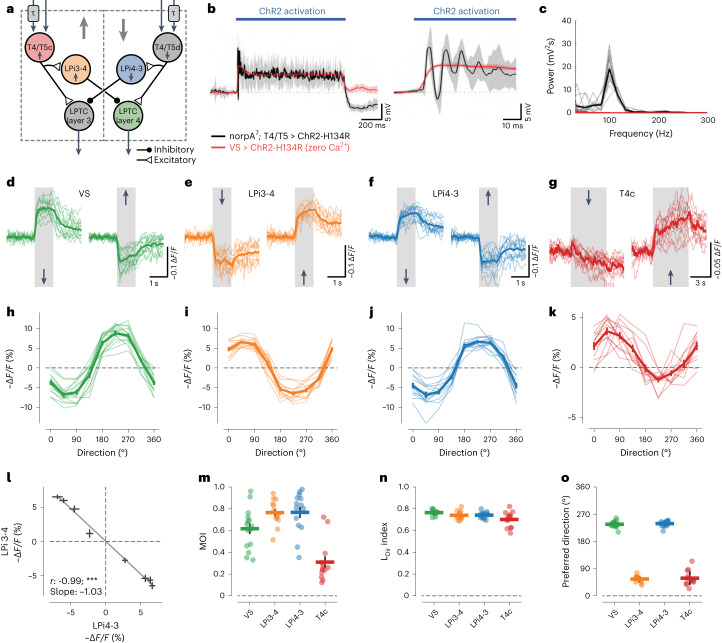
Motion-opponent voltage responses in the lobula plate motion vision circuitry. **a**, Neural circuit architecture of the core lobula plate circuitry. Differential temporal filtering of inputs to T4/T5 cells is indicated by the symbol τ. Of the four lobula plate layers, only layers 3 and 4 are shown for simplicity. **b**, Membrane potential responses of VS cells to optogenetic stimulation of either T4/T5 (black trace) or VS cells (red trace). Note that synaptic transmission was silenced by using Ca^2+^-free external saline when stimulating VS cells. The full response trace (left) and zoom-in (right) are shown. ChR2, channelrhodopsin-2. **c**, Power spectra of optogenetically induced responses from **b**. **d**–**g**, Voltage response traces of VS (**d**), LPi3-4 (**e**), LPi4-3 (**f**) and T4c cells (**g**) to gratings (**d**–**f**) or dots (**g**) moving in the PD or ND. **h**–**k**, Directional tuning curves for VS (**h**), LPi3-4 (**i**), LPi4-3 (**j**) and T4c cells (**k**). **l**, Linear regression between LPi3-4 and LPi4-3 cell voltage responses to the same stimulus directions. ***P  < 0.001. **m**–**o**, MOIs (**m**), L_Dir_ indices (**n**) and preferred tuning directions (**o**) of all imaged cell types. The data in **b**,**c** are from *n* = 3 flies per genotype. The data in **d**–**o** are from VS (*n* = 15), LPi3-4 (*n* = 13), LPi4-3 (*n* = 15) and T4c cells (*n* = 13 flies). The thin lines and dots represent individual flies. The thick lines and error bars indicate the mean ± s.e.m. In **l**, a Wald test was used. See also Extended Data Figs. 1 and 2. Source data

In this study, we took advantage of the experimental accessibility and electron microscopy (EM)-connectomic reconstructions of the *Drosophila* nervous system to investigate opponent inhibitory connections in the lobula plate motion vision circuitry. The major input elements to the lobula plate are small-field T4/T5 cells, which are the first direction-selective neurons in the ON (T4) and OFF (T5) pathways of the *Drosophila* visual system^11^. They come in four subtypes, each of which responds preferentially to one of the four cardinal directions of motion and sends axonal projections to a specific lobula plate layer. There, large-field lobula plate tangential cells (LPTCs) integrate excitatory input from multiple T4/T5 cells, thus inheriting their directional preference. T4/T5 cells also target inhibitory lobula plate-intrinsic (LPi) neurons, which in turn project onto LPTCs in the neighboring, oppositely tuned layer^12–14^ (Fig. 1a). This feedforward network is highly convergent: a single LPTC pools synaptic input from up to 700 T4/T5 cells and from around 7–8 LPi cells, whereas a single LPi cell collects input from up to 100 T4/T5 cells. LPTCs thus depolarize when confronted with stimuli moving along the preferred direction (PD) of presynaptic T4/T5 cells and hyperpolarize when stimuli move in the opposite, that is, null direction (ND)—a response property that is termed ‘motion-opponent’^15,16^. By integrating input from different subsets of T4/T5 cells, individual LPTCs are tuned to particular optic flow fields that match specific patterns of self-motion of the fly^17,18^. The additional inhibitory layer of LPi cells increases flow field selectivity by disambiguating flow fields that share motion components in one, but not the other direction^12^. This information about complex visual features is then used by downstream circuits to guide appropriate behavioral reactions^19–22^.

In this study, we reveal that a single cell type, LPi neurons, is implementing opponent inhibition at all three consecutive levels of the motion vision circuit: (1) at the axon terminals of T4/T5 cells; (2) by reciprocal inhibition between LPi cells themselves; and (3) at the dendrites of LPTCs. Our results suggest that the opponent stage at the level of LPTCs is essential to generate flow field selectivity, whereas motion opponency at the level of T4/T5 cells and LPi cells enhances the responsiveness of LPTCs under noisy conditions.

## Results

### Inhibitory feedback connections in the lobula plate circuitry

How motion-opponent visual responses in vertical system (VS) cells—an LPTC subtype—arise by LPi-mediated feedforward inhibition was demonstrated previously^12^ (Fig. 1a). While exploring synaptic transmission from T4/T5 to VS cells^13^, we observed that prolonged optogenetic stimulation of all T4/T5 cells induced fast membrane potential oscillations in postsynaptic VS cells (Fig. 1b). These oscillations were absent when directly stimulating synaptically isolated VS cells (Fig. 1b) and occurred with a frequency of approximately 100 Hz (Fig. 1c); thus, they are indicative of fast synaptic feedback inhibition in the lobula plate^23,24^. Therefore, we aimed to identify the anatomical basis and physiological function of these putative inhibitory feedback connections.

### Motion-opponent voltage responses in lobula plate neurons

**Extended Data Fig. 1:**
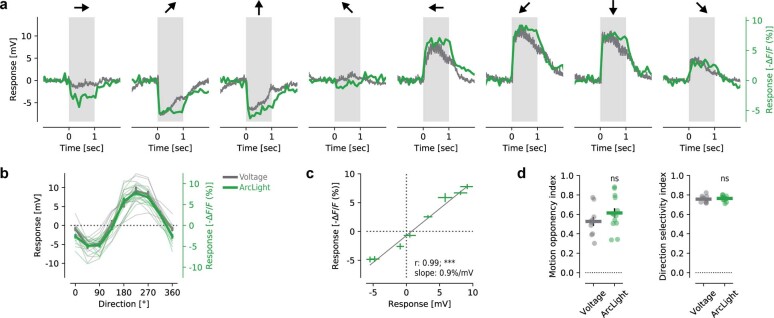
Voltage imaging reliably reports motion-opponent visual responses in VS Cells. **a**, Comparison of VS cell responses to gratings moving in eight different directions measured either electrophysiologically (grey) or with two-photon voltage imaging (green). **b**, Directional tuning curves of VS cell responses from data shown in (**a**). **c**, Linear regression between electrophysiological and voltage imaging responses of VS cells to same stimulus directions. **d**, Motion opponency index (left) and direction selectivity index (right) for data measured electrophysiologically or with voltage imaging. Data in (**a-d**) are from n = 5 flies, 10 cells (electrophysiology) and n = 7 flies, 13 cells (voltage imaging). Response traces: mean responses. Thin lines or dots: individual cells. Bars and error bars: mean ± s.e.m. Statistical tests: Wald test (**c**) and two-sided unpaired Student’s t-test (**d**). Source data

To explore the sources and tuning of inhibition in the lobula plate motion vision circuit, we performed two-photon voltage imaging of all key neural circuit components (Fig. 1a) in response to visual motion using the genetically encoded voltage indicator ArcLight^25^ (Extended Data Fig. 1). We first confirmed motion-opponent voltage responses and directional tuning when imaging the dendrites of VS cells (Fig. 1d,h). Next, we imaged voltage responses in LPi3-4 cells, which are tuned to upward motion based on their calcium signals^12^. In agreement with calcium imaging, LPi3-4 cells depolarized to upward motion. When stimulated with downward motion, LPi3-4 cells responded with a strong sustained hyperpolarization (Fig. 1e,i). Similarly, the oppositely tuned LPi4-3 cells depolarized to their PD (downward) and hyperpolarized to their ND (upward) (Fig. 1f,j). A comparison of the directional tuning curves of both LPi neuron types revealed mirror-symmetrical tuning properties (Fig. 1l). Thus, LPi cells—which account for the motion opponency of VS cells^12^—are themselves already fully motion-opponent.

**Extended Data Fig. 2:**
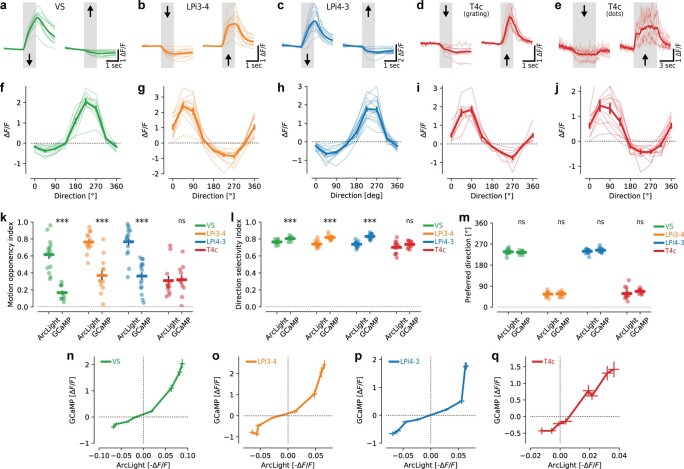
Motion-opponent calcium responses in the lobula plate motion vision circuitry. **a-e**, Calcium responses of VS (**a**), LPi3-4 (**b**), LPi4-3 (**c**) and T4c cells (**d,e**) to gratings (**a-d**) or dots (**e**) moving in the preferred- or null-direction. **f-j**, Directional tuning curves for VS (**f**), LPi3-4 (**g**), LPi4-3 (**h**) and T4c cells (**i,j**). **k-m**, Motion opponency indices (**k**), direction selectivity indices (**l**) and preferred directions (**m**) calculated from voltage responses (ArcLight) or calcium responses (GCaMP). Voltage imaging data in (**k-m**) are replotted from Fig. 1m–o. **n-q**, Plotting calcium against voltage responses of VS (**n**), LPi3-4 (**o**), LPi4-3 (**p**) and T4c cells (**q**) reveals a rectifying transformation between these two signals. Calcium imaging data in (a-m) are from VS: n = 11, LPi3-4: n = 13, LPi4-3: n = 11, T4c (grat): n = 6 and T4c (dots): n = 10 flies. Data in (**n-q**) are same as in (**f,g,h,j**) for calcium response and Fig. 1h–k for voltage responses. Thick lines in (**a-j**): mean data, thin lines: individual flies. Data in (**f-q**): mean ± s.e.m. Dots in (**k-m**): individual flies. Statistical test in (**k-m**): two-sided unpaired Student’s t-test. Source data

We then performed voltage imaging of T4c cell axon terminals, which arborize in layer 3 of the lobula plate and are tuned to upward motion. T4c terminals depolarized in response to upward motion and hyperpolarized, albeit less strongly, to downward motion, indicating that they also receive motion-opponent inhibitory input (Fig. 1g,k). We quantified motion-opponent responses by calculating a motion opponency index (MOI) (Fig. 1m). Motion opponency was highest in LPi neurons followed by VS cells. T4 cells showed lower levels of motion opponency on average. We further quantified each cell type’s direction selectivity (L_Dir_) and preferred tuning direction (Fig. 1n,o). Additionally, to gauge the transformation from voltage to calcium, we measured calcium responses in the above cell types to the same stimuli (Extended Data Fig. 2). All cell types again responded in a motion-opponent manner; however, with the exception of T4c cells, MOIs were much lower when comparing calcium with voltage, arguing for a nonlinear, rectifying voltage-to-calcium transformation (Extended Data Fig. 2k,n–q). In contrast, L_Dir_ was generally higher for calcium responses (Extended Data Fig. 2l). Preferred tuning directions were not different between voltage and calcium recordings (Extended Data Fig. 2m).

### Input from the oppositely tuned layer generates motion opponency

**Fig. 2:**
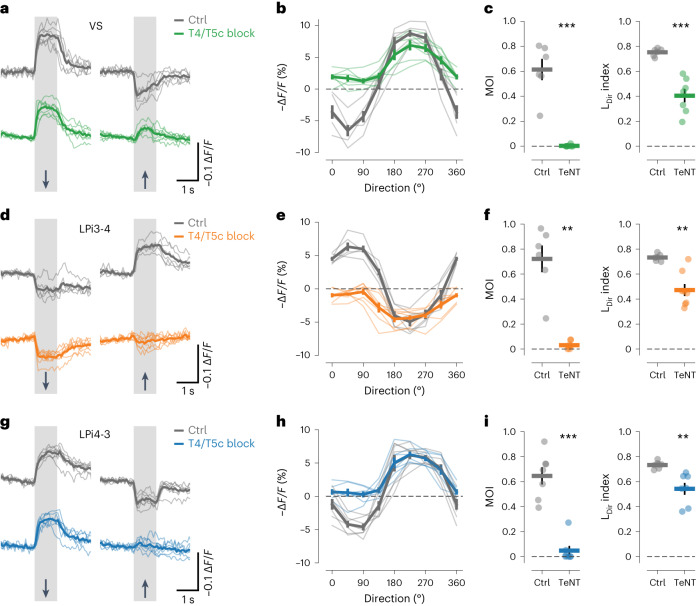
Direction-selective input from the oppositely tuned lobula plate layer generates motion opponency. **a**–**c**, Voltage response traces to PD and ND motion (**a**), directional tuning curves (**b**) and motion opponency (left) and L_Dir_ indices (right) (**c**) of VS cells from control (gray) and T4/T5c block flies (color). **d**–**f**, Same as **a**–**c** but for LPi3-4 cells. **g**–**i**, Same as **a**–**c** but for LPi4-3 cells. The data in **a**–**c** are from T4/T5c block (*n* = 7) and Ctrls (*n* = 6 flies). The data in **d**–**f** are from T4/T5c block (*n* = 7) and Ctrls (*n* = 6 flies). The data in **g**–**i** are from T4/T5c block (*n* = 7) and Ctrls (*n* = 7 flies). The thin lines and dots represent individual flies. The thick lines and error bars indicate the mean ± s.e.m. In **c**,**f**,**i**, a two-sided Welch’s *t*-test was used. **P < 0.01, ***P < 0.001. Source data

We next sought to identify the origins of motion-opponent responses in LPi cells, which could in principle arise according to two distinct mechanisms. First, LPi cells could inherit motion opponency directly from presynaptic T4/T5 cells. Second, LPi cells could receive direct depolarizing input from T4/T5 cells with aligned tuning and indirect hyperpolarizing input from oppositely tuned T4/T5 cells via yet unidentified cross-inhibitory connections. To directly test the latter hypothesis, we blocked synaptic transmission from upwardly tuned T4/T5c cells by expressing tetanus neurotoxin (TeNT) while measuring voltage responses to motion stimuli in LPi3-4, LPi4-3 and VS cells. Silencing the synaptic output of T4/T5c cells completely abolished ND hyperpolarization to upward motion in VS cells, in agreement with an indirect input from T4/T5c cells that is sign-inverted by inhibitory LPi3-4 cells (Fig. 2a,b). LPi3-4 cells, in turn, lost depolarizing responses to upward motion when blocking the direct excitatory input from T4/T5c cells (Fig. 2d,e). LPi4-3 cells showed the same effect on blocking of T4/T5c cells as VS cells—hyperpolarizing responses to upward motion were absent (Fig. 2g,h). Thus, silencing upwardly selective T4/T5c cells abolished the responses of downstream cells to upward motion, whereas responses to the opposite direction were left untouched. Thus, motion opponency was lost in all cell types and L_Dir_ was reduced (Fig. 2c,f,i). We conclude that fully motion-opponent responses in LPi and VS cells arise de novo by integrating output from oppositely tuned T4/T5 cells across neighboring lobula plate layers.

### Connectomics reveals a multilevel motion-opponent network

**Fig. 3:**
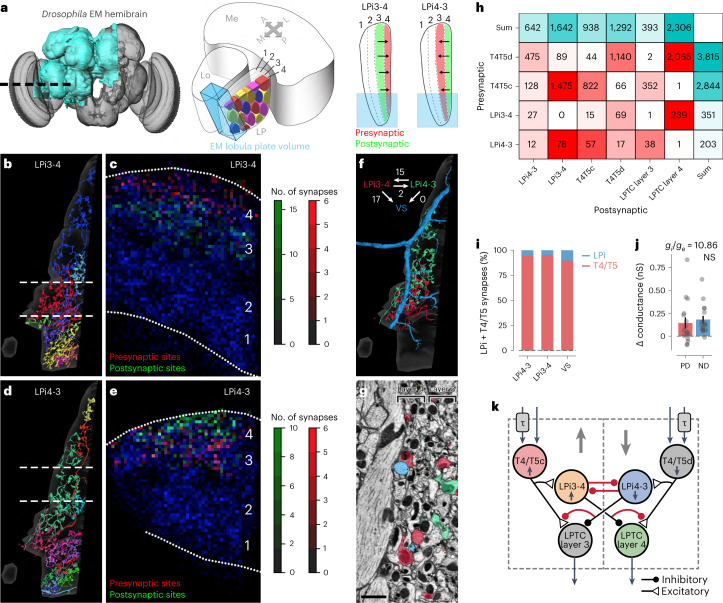
Connectomic analysis of the lobula plate motion vision network. **a**, Schematic of the *Drosophila* hemibrain volume (left). A part of the lobula plate is included in the reconstructed EM volume (middle, indicated in light blue). LPi3-4 cells are mainly presynaptic in layer 4 and postsynaptic in layer 3 of the lobula plate, while the opposite is true for LPi4-3 cells (right). **b**,**c**, Reconstructed LPi3-4 cells (partly fragmented) from the hemibrain dataset (**b**). Presynaptic (red) and postsynaptic (green) sites of LPi3-4 cells in relation to all synapses (blue) were detected in the corresponding EM volume (indicated with dashes in **c**). Relative positions of the lobula plate layers are indicated with numbers 1–4. **d**,**e**, Same as **b**,**c** but for LPi4-3 cells. **f**, Example of single LPi3-4, LPi4-3 and VS cells and their synaptic connections. **g**, Single EM section with cell profiles from cells in **f** highlighted in color. Scale bar, 2 µm. **h**, Connectivity matrix of the analyzed cell types. **i**, Percentage of synapses from T4/T5 and LPi cells onto other LPi or VS cells. **j**, VS cell conductance change to PD or ND visual stimulation (Extended Data Fig. 3). NS, not significant. **k**, Updated schematic of the lobula plate network. Newly identified synaptic connections are highlighted in red. Data in **j**: *n* = 16 VS cells. The dots indicate individual cells. The bars and error bars indicate the mean ± s.e.m. The statistical test used was a two-sided Wilcoxon signed-rank test. Source data

The finding that motion opponency in LPi cells arises by integrating signals across neighboring lobula plate layers (Fig. 2) prompted us to identify the synaptic connections that mediate cross-inhibition between layers. To this end, we performed analysis of synaptic connectivity in the lobula plate by taking advantage of a connectome obtained from dense reconstruction of EM data^26^. The motion-opponent responses of LPi3-4 and LPi4-3 cells could plausibly be explained by inhibitory cross-connectivity between these cell types themselves. We thus asked whether such a connectivity pattern is apparent in the hemibrain connectome^26^ (Fig. 3a).

Starting our analysis from VS cells, we identified putative LPi3-4 and LPi4-3 cells according to the following criteria: (1) both are bistratified and resemble genetically defined cells in shape^12,27^ (Fig. 3b,d); (2) the presynaptic sites (T-bars) of LPi3-4 cells are largely restricted to layer 4 whereas the presynaptic sites of LPi4-3 cells are found predominantly in layer 3 (Fig. 3c,e); (3) LPi3-4 cells receive synaptic input from T4/T5c mainly in layer 3, whereas LPi4-3 cells obtain major T4/T5d input in layer 4; (4) LPi3-4 cells are strongly connected to VS cells^12,14^, whereas LPi4-3 cells provide input to yet uncharacterized layer 3 output neurons (Fig. 3f–h). As seen in the connectivity matrix (Fig. 3h), we found that the two LPi cell types were indeed reciprocally connected, providing an explanation of how their motion opponency arises. Furthermore, the connectome data suggested that LPi cells provide selective synaptic input to T4/T5 axon terminals with opposite directional tuning (Fig. 3h), probably explaining the fingerprints of motion opponency that we observed in T4c voltage and calcium measurements (Fig. 1g,k and Extended Data Fig. 2d,e). We also detected many T4-T4 and T5-T5 connections between cells of the same subtype (Fig. 3h), as reported earlier^28,29^. As these connections were almost exclusively between cells with the same directional tuning, we inferred that they do not contribute to motion-opponent computations and thus did not investigate them further. On the whole, our analysis is also consistent with a wiring diagram derived from a different EM dataset^14^.

**Extended Data Fig. 3:**
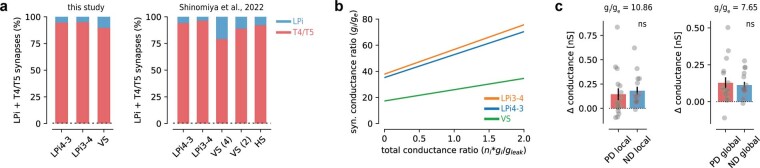
Inhibitory synapses have a much higher unitary conductance than excitatory synapses. **a**, Percentage of synapses from T4/T5 and LPi cells onto different other cell types. Data are from the present study (left) or a recent publication (right). VS (4) and VS (2) refers to dendritic branches of VS cells that arborize in lobula plate layer 2 or 4. **b**, Synaptic conductance ratio relative to total conductance ratio for different cell types, as calculated from a simple biophysical model. **c**, VS cell conductance change in response local (left) and global (right) transparent motion moving in the preferred- or null-direction (see also Fig. 7b–h). Synaptic conductance ratios (g_i_/g_e_) derived from the measurements are plotted above. Data in (**c**) is from n = 16 VS cells. Dots: individual cells. Bars and error bars: mean ± s.e.m. Statistical test in (**c**): two-sided Wilcoxon signed-rank test. Source data

Unexpectedly, the number of inhibitory LPi synapses onto VS or different LPi cells amounted to only between 5% and 20% of summed LPi and T4/T5 inputs in both datasets (Fig. 3i and Extended Data Fig. 3a). Given almost symmetrical voltage responses (Fig. 1h–j), a simple biophysical model predicted that the unitary synaptic conductance for glutamatergic inhibitory (LPi) synapses should be at least tenfold higher than for cholinergic excitatory (T4/T5) synapses (Extended Data Fig. 3b). Therefore, we used electrophysiology to measure conductance changes in VS cells to visual stimulation, which led to estimated inhibitory to excitatory conductance ratios $(g_{{\mathrm{i}}}/{g}_{{\mathrm{e}}})$ of around 7.7 and 10.9, in agreement with the model’s prediction (Fig. 3j and Extended Data Fig. 3c).

The newly identified synaptic connections called for a revised lobula plate wiring diagram in which single LPi cells are wired to inhibit T4/T5 axon terminals, LPi cells with opposite tuning and LPTC dendrites, thus performing motion-opponent computations at all three consecutive levels of the core lobula plate circuit (Fig. 3k).

### Motion-opponent inhibition is mediated by GluClα receptors

**Fig. 4:**
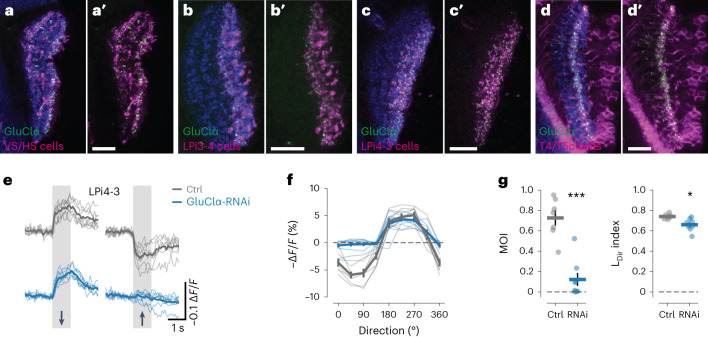
Motion-opponent inhibition is mediated by the glutamate-gated chloride channel GluClα. **a**,**a**′, The inhibitory glutamate receptor GluClα localizes to VS and HS cell dendrites. **b**,**b**′, GluClα localizes mainly to lobula plate layer 3 in LPi3-4 cells. **c**,**c**′, GluClα localizes predominantly to lobula plate layer 4 in LPi4-3 cells. **d**,**d**′, GluClα localizes to the axon terminals of T4/T5c cells in lobula plate layer 3. nc82 staining is shown in blue in **a**–**d**. **e**–**g**, Voltage response traces to PD and ND motion (**e**), directional tuning curves (**f**) and MOIs (left) and L_Dir_ indices (right) (**g**) of LPi4-3 cells from control (gray) and GluClα-RNAi flies (blue). Scale bars in **a**–**d**, 10 µm. Data in **e**–**g** are from Ctrls (*n* = 7) and GluClα-RNAi (*n* = 7 flies). *P < 0.05, ***P < 0.001. The dots and thin lines represent individual flies. The thick lines and error bars indicate the mean ± s.e.m. In **g**, a two-sided Welch’s *t*-test was used. See also Extended Data Fig. 4. Source data

**Extended Data Fig. 4:**
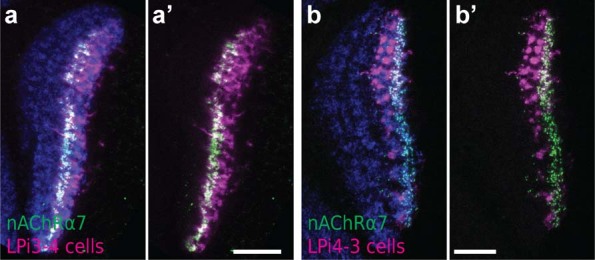
Subcellular localization of nAChRα7 nicotinic acetylcholine receptors in LPi cells. **a-a’**, nAChRα7 localizes mainly to lobula plate layer 3 in LPi3-4 cells. **b-b’**, nAChRα7 localizes predominantly to lobula plate layer 4 in LPi4-3 cells. Scale bars: 10 µm. Source data

The reciprocal synaptic connections between LPi4-3 and LPi3-4 neurons, their glutamatergic neurotransmitter profile^12,30^ and the predicted inhibitory nature of these synapses indicated that both LPi types express the inhibitory glutamate-gated chloride channel GluClα. Furthermore, the finding that LPi3-4 cells are mainly presynaptic in lobula plate layer 4 and LPi4-3 mainly in layer 3 (Fig. 3c,e) suggested that GluClα predominantly localizes to these layers in postsynaptic cells. As predicted, a GluClα-green fluorescent protein (GFP) reporter^31^ mainly localized to VS cell dendrites, to lobula plate layer 3 in LPi3-4 cells and to layer 4 in LPi4-3 cells (Fig. 4a–c). Additionally, the nicotinic acetylcholine receptor subunit Dα7 was detected in the same layers as GluClα, which is consistent with cholinergic input from T4/T5 cells (Extended Data Fig. 4). High levels of the GluClα receptor were also found on the axon terminals of T4/T5c cells^31^—in agreement with inhibitory feedback connections from LPi4-3 cells onto T4/T5c terminals (Fig. 4d). To confirm the functional role of GluClα in generating ND inhibition, we investigated the effect of GluClα-RNA interference (RNAi) knockdown on visual responses of LPi4-3 neurons as an exemplar (Fig. 4e–g). Depleting GluClα from LPi4-3 cells strongly diminished ND hyperpolarization, whereas PD depolarization was unaltered (Fig. 4e,f). This led to a strong reduction in motion opponency and decreased L_Dir_ (Fig. 4g). Thus, GluClα receptors mediate motion-opponent inhibition in LPi4-3 cells and probably also LPi3-4 cells, VS cells and T4/T5 terminals.

### Transparent motion reveals sources and strength of inhibition

**Fig. 5:**
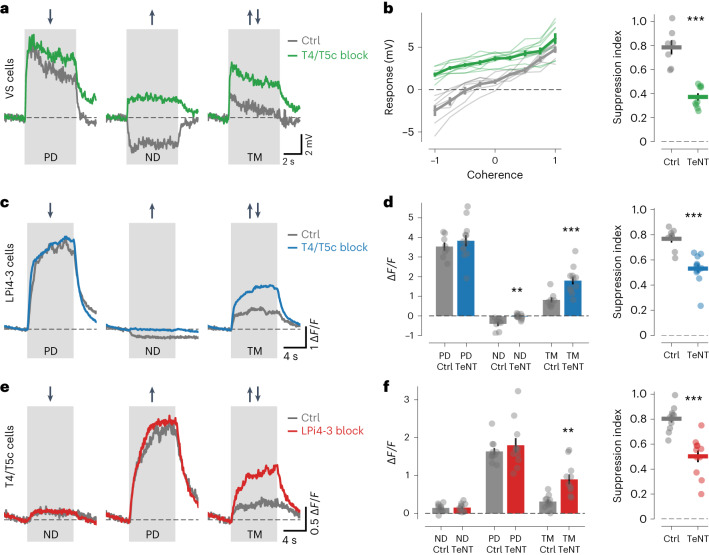
Voltage and calcium responses of VS, LPi4-3 and T4/T5c cells to TM. **a**,**b**, Voltage responses of VS cells to PD, ND and TM from control (gray) or T4/T5c block flies (green). Response traces are shown in **a**. Mean responses for different ‘coherences’ of moving dots (left) and motion-opponent suppression indices (right) are shown in **b**. **c**,**d**, Calcium responses of LPi4-3 cells to PD, ND and TM from Ctrl (gray) or T4/T5c block flies (blue). Response traces are shown in **c**. Bar plots of mean responses (left) and motion-opponent suppression indices (right) are shown in **d**. **e**,**f**, Same as in **c**,**d** but for calcium responses of T4/T5c cells from Ctrl (gray) and LPi4-3 block flies (the data in **a**,**b** are from Ctrl (7 cells) and T4/T5c block (9 cells); the data in **c**,**d** are from Ctrl (8 flies) and T4/T5c block (12 flies); and the data in **e**,**f** from Ctrl (11 flies) and LPi4-3 block (11 flies)). The voltage or calcium traces in **a**,**c**,**e** indicate the mean responses. The thin lines or dots in **b**,**d**,**f** represent individual cells or flies. The thick lines or bars in **b**,**d**,**f** indicate the mean ± s.e.m. The statistical test used was a two-sided Welch’s *t*-test. See also Extended Data Fig. 5. **P < 0.01, ***P < 0.001. Source data

**Extended Data Fig. 5:**
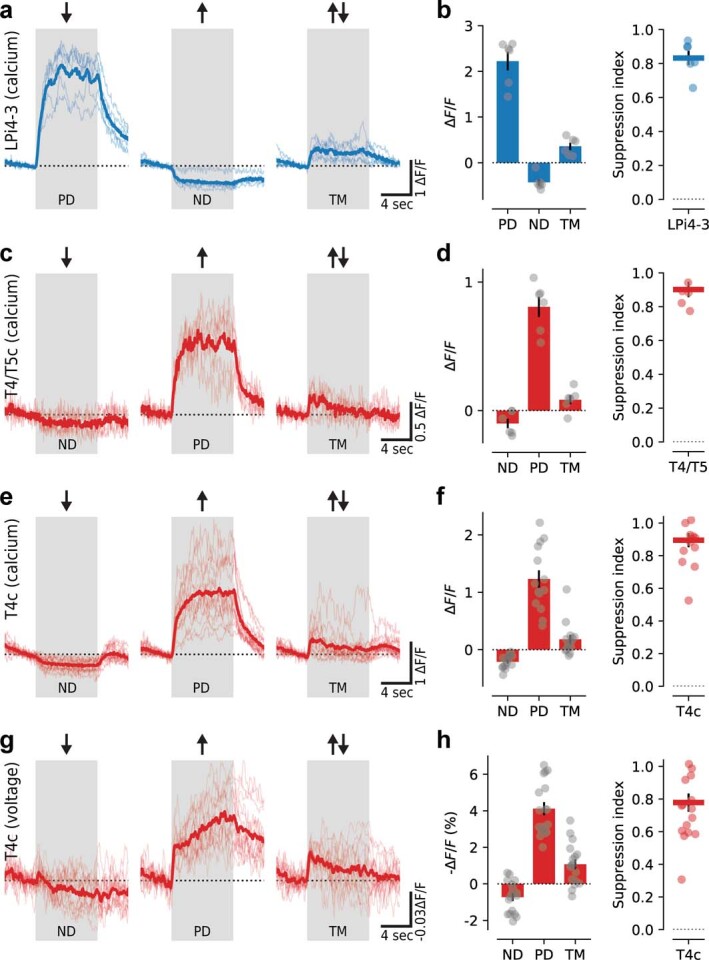
Voltage and calcium responses of LPi4-3, T4/T5c and T4c cells to transparent motion. **a,b**, Calcium responses of LPi4-3 cells to preferred-direction (PD), null-direction (ND) and transparent motion (TM). (**a**) Calcium response traces. (**b**) Bar plots (left panel) and motion-opponent suppression index (right panel). **c,d**, Similar to (**a,b**) but for T4/T5c cells instead of LPi4-3 cells. **e,f**, Similar to (**a,b**) but for T4c cells instead of LPi4-3 cells. **g,h**, Similar to (**e,f**) but for voltage responses of T4c cells instead of calcium responses. Data in (**a,b**) are from LPi4-3: 6 flies, in (**c,d**) from T4/T5c: 6 flies, in (**e,f**) from T4c: 14 flies, and in (**g,h**) from T4c: 17 flies. Thin lines or dots: individual flies. Thick lines: mean responses. Bars: mean ± s.e.m. Source data

**Extended Data Fig. 6:**
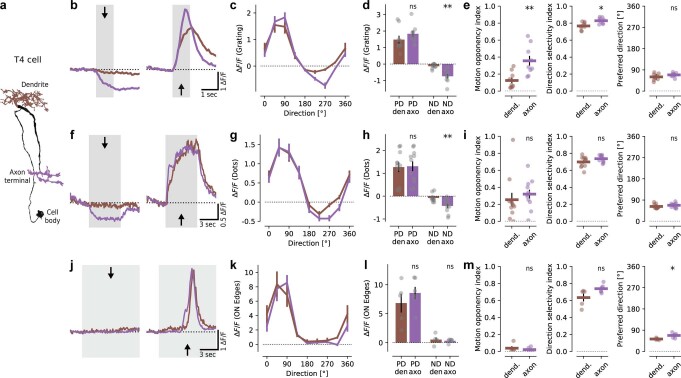
Axonal and dendritic calcium responses in T4c cells. **a**, Anatomy of a single T4 cell with dendrite, axon terminal and cell body indicated. **b**, Calcium responses of T4c cell dendrites (brown) and axons (violet) to sine-wave gratings moving in the null- (left) or preferred-direction (right). **c**, Directional tuning curves for T4c cell dendrites (brown) and axons (violet). **d**, Mean responses for preferred-direction (PD, 90°) and null-direction (ND, 270°) stimuli. **e**, Motion opponency (left), direction selectivity (middle) and preferred directions (right) of T4c cell dendrites and axons. **f-i**, Same as (**b-e**) but in response to dot motion stimuli. **j-m**, Same as (**b-e**) but in response to moving ON edges. Note that T4c cell dendrites and axons do not show differences in motion opponency and direction selectivity in response to moving ON edges. This is presumably because the blank arena screen before stimulus motion does not lead to elevated calcium levels (as for grating and dot stimuli), resulting in a floor effect. Data in (**b-e**) are from T4c dendrites: n = 9, T4c axons: n = 9 flies. Data in (**f-i**) are from T4c dendrites: n = 11, T4c axons: n = 11 flies. Data in (**j-m**) are from T4c dendrites: n = 5, T4c axons: n = 6 flies. Response traces in (**b,f,j**): mean responses. Thick lines and error bars: mean ± s.e.m. Dots: individual flies. Statistical tests: two-sided dependent t-test for paired samples (**d,e**) and (**h,i**) and two-sided unpaired Student’s t-test (**l,m**). Source data

To investigate motion-opponent inhibition in further detail, we used a transparent motion (TM) stimulus that contained balanced PD and ND motion components at the same time^32–34^ (Supplementary Video 1). The reduction in response to TM compared with the PD response allowed for an estimation of the strength of motion-opponent suppression. We first performed electrophysiological experiments in VS cells. Randomly placed dots moving coherently in the cells’ PD or ND led to depolarization and hyperpolarization, respectively (Fig. 5a). TM, that is, simultaneous motion of half of the dots in PD and the other half in ND, evoked only weak responses, indicative of strong motion-opponent suppression. When changing the fraction of dots that moved in the PD or ND, VS cell responses varied linearly with the fractional difference of dots moving in PD and ND (here termed ‘coherence’) (Fig. 5b). Calcium signals in LPi4-3 cells also showed motion-opponent suppression when confronted with TM (Fig. 5c,d and Extended Data Fig. 5a,b). Like in VS and LPi4-3 cells, strong motion-opponent suppression also occurred in T4/T5c and T4c axon terminals at the level of calcium (Fig. 5e,f and Extended Data Fig. 5c–f), confirming previous results^32^, and at the level of membrane voltage in T4c cells (Extended Data Fig. 5g,h). Furthermore, we observed hyperpolarization and calcium reduction in T4c terminals in response to ND motion (Extended Data Fig. 5e–h). Interestingly, the decrease in calcium levels was stronger in T4c axon terminals than in dendrites for multiple visual stimuli (Extended Data Fig. 6). Thus, both motion opponency and L_Dir_ were higher in T4c axons than in dendrites (Extended Data Fig. 6d,e), a further fingerprint of opponent inhibition impinging onto T4/T5 axon terminals.

Next, we removed LPi3-4-mediated inhibitory input to VS cells by blocking T4/T5c cells and presented PD, ND and TM stimuli. Whereas responses to PD were unaffected, hyperpolarization to ND stimulation was abolished in block flies, as expected when removing ND inhibition. Responses to TM were increased in comparison with control flies, revealing reduced motion-opponent suppression. However, opponent suppression was not completely lost, but still at a level of around 40% (Fig. 5a,b). Performing analogous experiments in LPi4-3 cells using calcium imaging gave similar results—loss of ND responses and reduced motion-opponent suppression when blocking T4/T5c cells (Fig. 5c,d). We then investigated the origins of motion-opponent suppression in T4/T5 cells. A previous study suggested that motion-opponent suppression in T4/T5 cells arises exclusively on their dendrites because of the interaction between dendritic input elements^32^, as would be predicted by a three-arm motion detector model^35–38^. However, our experiments indicated that T4/T5 cells also received axonal inhibition from oppositely tuned LPi cells. To test this synaptic connection functionally, we blocked LPi4-3 cells and imaged calcium responses in T4/T5c terminals. Interestingly, motion-opponent suppression was still present in LPi block flies; however, it was substantially reduced compared to controls, as in the experiments in VS and LPi4-3 cells (Fig. 5e,f). These results suggest that two complementary mechanisms generate motion-opponent suppression in T4/T5 cells: a dendritic mechanism, arising from the interaction of dendritic inputs and unaffected by blocking LPi cells, and an axonal mechanism that is dependent on inhibitory input from LPi cells.

### A spatiotemporal gradient across lobula plate circuit levels

**Fig. 6:**
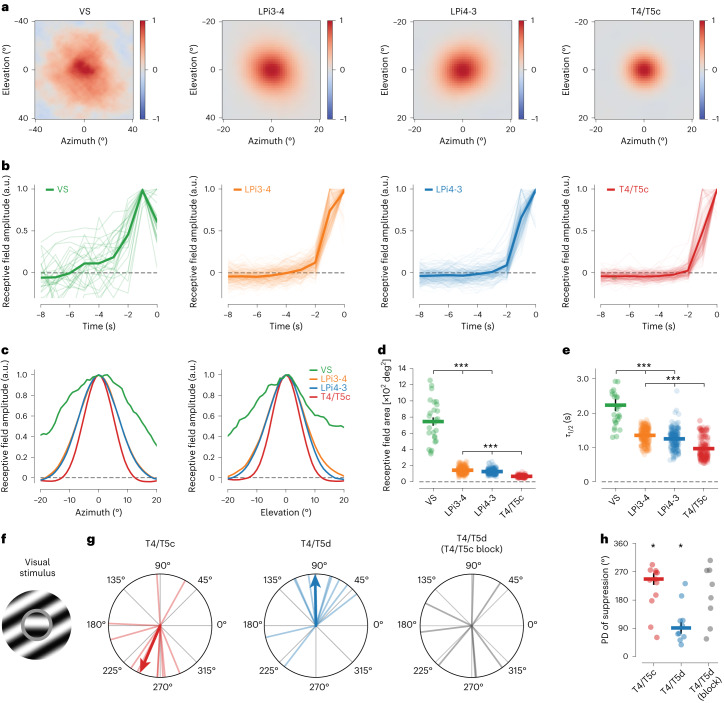
A gradient of spatiotemporal motion receptive fields in the lobula plate. **a**, Average spatial receptive fields of VS, LPi3-4, LPi4-3 and T4/T5c cells measured with stochastic motion noise. Spatial receptive fields correspond to the time point of maximal responses in **b**. Note the different scale of the *x* and *y* axes for VS cells compared with the other cell types. **b**, Temporal receptive fields (calculated from the spatial receptive field centers) of all cell types. **c**, One-dimensional slices through the centers of the spatial receptive fields from **a** along the azimuth (left) and elevation (right). **d**, Comparison of spatial receptive field areas. **e**, Comparison of the decay times (*τ*_1/2_) of the temporal receptive fields. **f**–**h**, Directional suppression of T4/T5 cells to an annulus motion stimulus. **f**, Schematic of the annulus motion stimulus (Methods). **g**, PD of suppression for T4/T5c (left) and T4/T5d (middle) cells, and T4/T5d cells with T4/T5c blocked (right). **h**, Comparison of PDs of suppression. Directional tuning of suppression is lost in T4/T5d cells on blocking T4/T5c cells. Data in **a**–**e** are from VS (*N* = 10, *n* = 26), LPi3-4 (*N* = 10, *n* = 237), LPi4-3 (*N* = 11, *n* = 199) and T4/T5c (*N* = 9, *n* = 201, with *N* = flies and *n* = regions of interest (ROIs). Data in **g**,**h** are from T4/T5c (*n* = 12), T4/T5d (*n* = 10) and T4/T5d (T4/T5c-block) (*n* = 8 flies). The thick lines indicate mean data. The thin lines or dots indicate individual ROIs. The data in **d**,**e**,**h** indicate the mean ± s.e.m. In **d**,**e**, a two-sided Holm’s-corrected Mann–Whitney *U*-test was used. In **h**, a Rayleigh *z*-test was used. *P < 0.05, ***P < 0.001. Source data

We identified distinct levels of motion-opponent computation at every stage of the lobula plate circuit. Why does this network perform a seemingly redundant operation on multiple consecutive instances? Based on the anatomical sizes of the cell types, we reasoned that the distinct opponent levels might have different spatiotemporal properties. We therefore characterized the spatiotemporal receptive fields of all cell types using a stochastic motion noise stimulus followed by reverse correlation^39^ and focused on individual axons or axonal boutons. VS cells had large receptive fields^18,40^ and slow temporal dynamics (Fig. 6a–e). LPi3-4 and LPi4-3 cells displayed intermediate spatial and temporal integration properties (Fig. 6a–e). The spatial receptive fields of single boutons from both LPi types were smaller than expected if cells were to integrate linearly over their complete arbor, pointing toward some extent of compartmentalized signaling. Lastly, T4/T5 terminals had the smallest receptive fields with the fastest temporal dynamics (Fig. 6a–e).

In line with the different spatial receptive field sizes of T4/T5 and LPi cells, calcium signals in T4/T5 axon terminals were subject to motion-opponent suppression when stimulated with a PD stimulus in the receptive field center while showing gratings moving in different directions in a surrounding annulus that was tailored to the size of LPi receptive fields. This motion-opponent suppression was dependent on synaptic output from T4/T5 cells with opposite directional preference (Fig. 6f–h), further supporting inhibitory input to T4/T5 cells from the neighboring lobula plate layer. In summary, we found a gradient of response properties from fast, small-field T4/T5 cells, over intermediate LPi cells to slow, large-field LPTCs (Fig. 6d,e). These differences in spatiotemporal integration might be key in understanding the functional roles of the different levels of opponency.

### Functional roles of multilevel motion-opponent inhibition

**Fig. 7:**
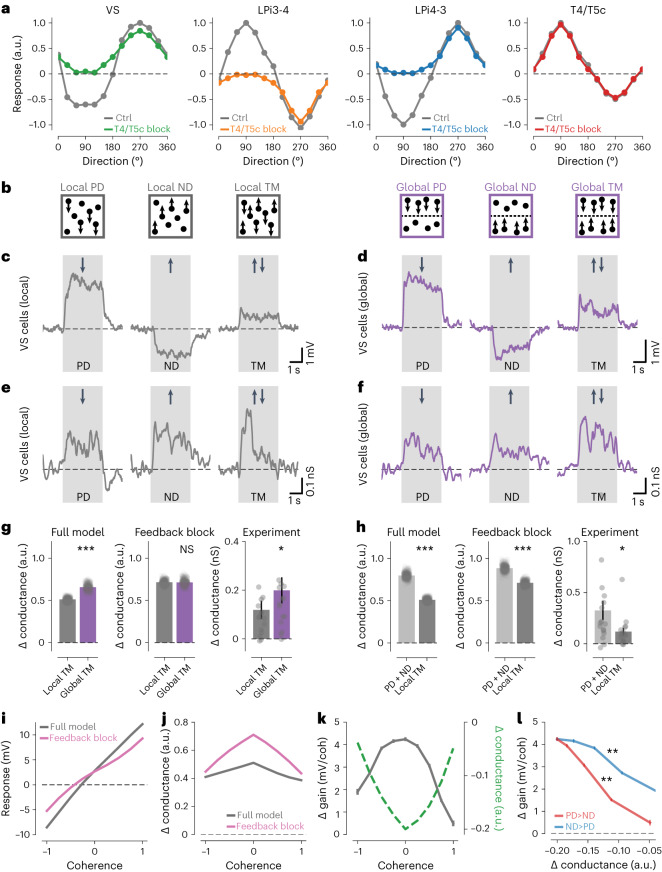
Functional roles of motion-opponent levels in the lobula plate. **a**, Directional tuning curves of model VS, LPi3-4, LPi4-3 and T4/T5c cells from lobula plate network simulations stimulated with moving gratings. The simulations were run with normal network connectivity (gray) or with T4/T5c cells blocked (colors). **b**, Cartoons illustrating the visual stimuli used below. **c**,**d**, Electrophysiologically measured voltage responses of VS cells to dots moving in PD, ND or to TM stimuli. ‘Local’ stimuli are shown in **c** (gray) and ‘global’ stimuli are shown in **d** (purple). **e**,**f**, Same as **c**,**d** but for electrophysiologically measured conductance changes instead of voltage responses. **g**, Conductance changes of full model (left) or when LPi-T4/T5 and LPi-LPi connections were blocked simultaneously (‘feedback block’, middle) and experimentally measured responses (right) for local (dark gray) and global (purple) TM stimuli. **h**, Like **g** but comparing the sum of local PD + ND (light gray) and local TM (dark gray). Note that, to facilitate comparison between experiment and model, 3 of 32 (**g**) and 1 of 32 (**h**) data points are beyond the arbitrarily set *y* axis limits. Plots with all individual data points are shown in Extended Data Fig. 8a–c. **i**,**j**, Voltage responses (**i**) and conductance changes (**j**) of VS cells from full network model (gray) or model without inhibitory feedback connections (pink) to local TM stimuli with different coherences (coh). **k**, Gain (gray) and conductance (green) differences between full model and feedback block for different motion coherences. **l**, Correlation between conductance difference and gain difference for PD-dominated (red, *r* = −0.99) or ND-dominated (blue, *r* = −0.98) motion. Data in **c**–**h**: *n* = 16 VS cells. Response traces indicate mean responses. The dots indicate individual cells or model runs. The bars and error bars indicate the mean ± s.e.m. A two-sided paired Student’s *t*-test was used for the model data in **g**,**h**; a two-sided Wilcoxon signed-rank test was used for the experimental data in **g**,**h**; and in **l**, a Wald test was used. See also Extended Data Figs. 7 and 8. *P < 0.05, **P < 0.01, ***P < 0.001. Source data

**Extended Data Fig. 7:**
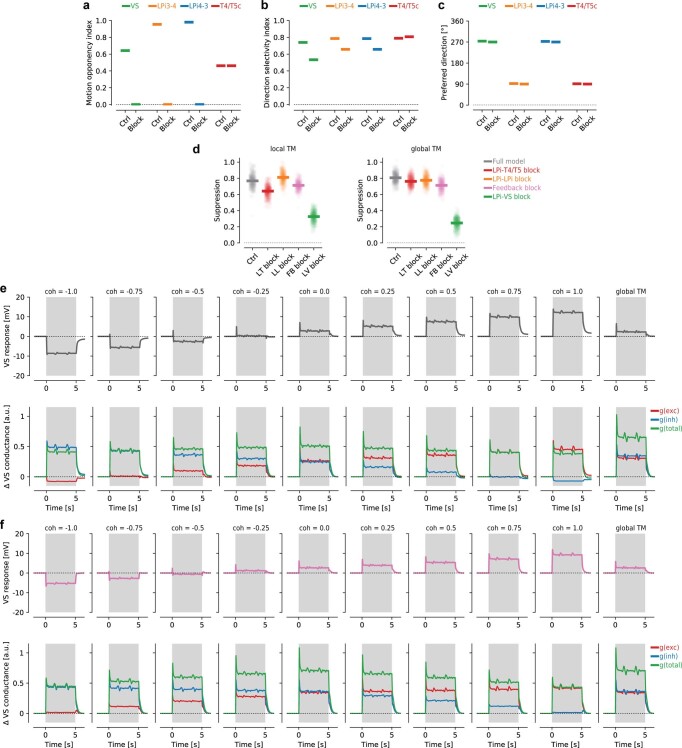
Tuning indices, voltage responses and conductance changes of the network model. **a-c**, Motion opponency indices (**a**), direction selectivity indices (**b**) and preferred directions (**c**), in response to sine-wave grating stimuli for different cell types of the full network model (Ctrl) and when blocking output from T4/T5c cells (Block, related to Fig. 7a). **d**, Response selectivity, as measured by motion-opponent suppression to the local (left) or global (right) transparent motion stimulus, for the full network model and when blocking the different motion-opponent levels. ‘Feedback block’ indicates that LPi-T4/T5 and LPi-LPi connections were blocked simultaneously. **e**, Voltage responses (upper panels) and conductance changes (lower panels) of model VS cells from the full network model to local transparent motion stimuli (local TM) with different coherences and to global transparent motion (global TM). **f**, Similar to (**e**) but when blocking inhibitory feedback-connections (‘feedback block’) in the network model. Data in (**a-c**) are from a single model run. Dots and thick lines in (**d**) represent individual model runs and mean values, respectively. Data in (**e-f**) represent mean values from 300 model runs. Source data

To synthesize our experimental findings, we constructed a computational model of the lobula plate network (Methods). Probing either the full model, or when blocking T4/T5c output connections, with moving gratings led to directional tuning curves that closely resembled the experimentally measured ones (Fig. 7a). L_Dir_, motion opponency and preferred tuning directions also agreed well with the experiments (Extended Data Fig. 7a–c). Additionally, model VS cells depolarized and hyperpolarized to PD and ND dot motion and exhibited strongly suppressed responses to TM, similar to the experiments (Extended Data Fig. 7e).

Next, we blocked inhibitory connections at each opponent level individually. Response selectivity, as measured by motion-opponent suppression to TM stimuli, was strongly reduced when blocking synapses between LPi and VS cells, but only mildly when blocking LPi-T4/T5, LPi-LPi or both types of connections together (Extended Data Fig. 7d). Thus, the earlier opponent levels operating at smaller (i.e. more local) spatial scales did not strongly affect response selectivity of the network, suggesting that they have different functional roles. In model VS cells, the suppression of excitatory input under TM stimulation is caused by an equally strong opposing inhibitory conductance, which leads to a profound increase in total membrane conductance (Extended Data Fig. 7e). This increased membrane conductance shunts additional incoming signals, thus effectively decreasing the gain of the neuron^41^. These considerations inspired a functional hypothesis: performing opponent inhibition already at earlier circuit levels, and thus at more local spatial scales (Fig. 6c,d), might partially relieve the VS cell membrane from impinging excitatory and inhibitory inputs caused by locally opponent motion noise. (In this study, noise is defined as everything that leads to simultaneous activation of oppositely tuned motion detectors and is thus noninformative about the direction of local image motion.)

**Extended Data Fig. 8:**
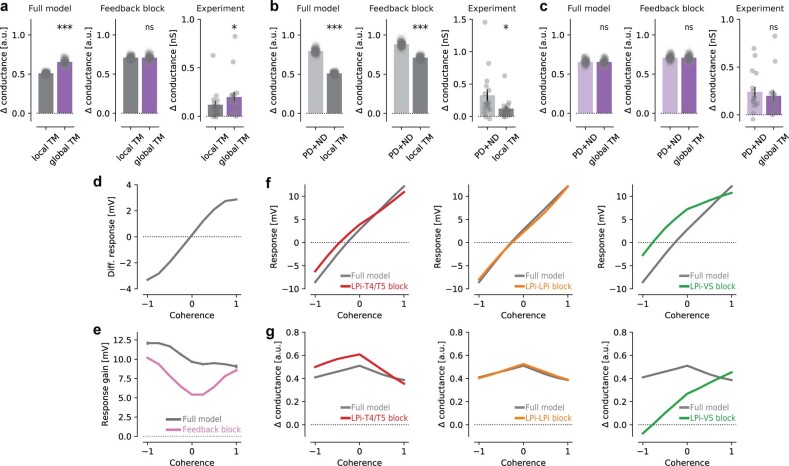
Additional results of the lobula plate network model. **a**, Conductance changes of network models with (left) or without feedback connections (middle) and experimentally measured responses (right) for local (dark grey) and global (dark purple) transparent motion stimuli (same data as Fig. 7g). **b**, Similar to (**a**) but comparing sum of local PD + ND (light grey) and local TM (dark grey), (same data as Fig. 7h). **c**, Similar to (**a**) but comparing sum of global PD + ND (light purple) and global TM (dark purple). **d**, Differential voltage responses between full network model and model without inhibitory feedback connections to local transparent motion stimuli with different coherences. **e**, Response gain of VS cells from full model (grey) and with feedback connections blocked (pink) for different motion coherences. **f,g**, Voltage responses (**f**) and conductance changes (**g**) of VS cells from full network model (grey) or models in which different inhibitory connections (red, LPi-T4/T5 block; orange, LPi-LPi block; green, LPi-VS block) were blocked in response to different motion coherences. Data in (**a-c**): n = 16 VS cells. Response traces: mean responses. Dots: individual cells or model runs. Bars and error bars: mean ± s.e.m. Statistical tests: two-sided paired Student’s t-tests (model data) and two-sided Wilcoxon signed-rank tests (experimental data). Source data

We tested this hypothesis by investigating three predictions that follow from it: (1) first, the conductance change in VS cells should be higher for a stimulus that contains motion components that are only globally opponent (i.e. at the spatial scales of the receptive fields of VS cells), but not locally opponent (i.e. at the spatial scales of the receptive fields of T4/T5 and LPi cells). This requirement is fulfilled by a stimulus in which all dots on one half of the screen move in the PD and all dots on the other half move in the ND, which is reminiscent of an optic flow field caused by forward translation (‘global TM’; Fig. 7b and Supplementary Video 2). Conversely, the TM stimulus used in previous experiments (‘local TM’; Figs. 5 and 7b and Extended Data Fig. 5) also contains locally opponent motion components (i.e. local motion noise). Fly VS cells responded similarly to ‘local’ and ‘global’ TM stimuli at the level of membrane voltage (Fig. 7c,d). However, conductance changes in model VS cells revealed a significant difference in response to local and global TM, in line with our first prediction (Fig. 7g, left). To test whether the earlier, locally opponent levels caused this effect, we blocked inhibitory feedback connections from LPi cells to T4/T5 cells and between LPi types (‘feedback block’) in the model. Consequently, conductance changes in response to locally and globally opponent stimuli were rendered indistinguishable (Fig. 7g, middle; Extended Data Fig. 7f). We then tested prediction (1) with conductance measurements in VS cells (Fig. 7e,f). Conductance changes in response to global TM in VS cells were indeed around 1.7-fold higher than to local TM, supporting our functional hypothesis (Fig. 7g and Extended Data Fig. 8a); (2) a second prediction was that the conductance change to local TM should be smaller than the sum of conductance changes caused by its individual PD and ND motion components, and this effect should again depend on the presence of inhibitory feedback connections. Model simulations were largely in agreement with this prediction, although blocking feedback connections only led to a reduction, not to full abolishment, of the predicted effect (Fig. 7h). Importantly, the experimentally measured conductance change in VS cells to local TM was indeed smaller than the sum of PD and ND motion components measured in isolation, and this effect was even more pronounced than predicted by the full network model (Fig. 7h, right; Extended Data Fig. 8b); (3) as a third prediction, the conductance change for the global TM stimulus should be equal to its individual components, independent of the presence of inhibitory feedback. Network simulations and electrophysiological measurements were congruent with this prediction (Extended Data Fig. 8c), further corroborating our functional hypothesis.

### Local motion opponency enhances neural network response gain

Lastly, we extended our computational model to explore the relationship between locally opponent noise and VS cell responses in more detail. We varied the relative amounts of PD and ND motion components (i.e. coherence), and thus the amount of motion opponency, and compared VS cell responses and conductance changes between the full model and when inhibitory connections were blocked (Fig. 7i–l and Extended Data Fig. 8d–g). Blocking opponent feedback inhibition led to a reduction of response strength over all coherences (Fig. 7i and Extended Data Fig. 8d). Importantly, blocking feedback connections also led to a pronounced reduction in response gain, measured as the slope of the response curve (Fig. 7k and Extended Data Fig. 8e), whereas the total change in conductance increased for all coherences (Fig. 7j). Comparing differences in response gain and conductance between full model and feedback block revealed an inverse relationship (Fig. 7k). The difference in response gain was highest when opponency was highest (i.e. at coherence = 0), which was exactly when VS cell conductance was most strongly reduced. Response gain and conductance were thus anticorrelated for both PD- and ND-dominated motion (Fig. 7l). Together, these results suggest that the motion-opponent levels discovered in this study function to reduce the conductance load on VS cell dendrites when locally opponent noise is high, which enhances the sensitivity of the network under such conditions.

In conclusion, motion-opponent inhibition at the level of T4/T5 terminals and LPi cells suppresses locally opponent signals, whereas the VS cell level cancels out stimuli that are globally opponent. This network architecture enables the circuit to be resilient to locally opponent noise while enabling global flow field selectivity with high gain.

## Discussion

In this study, we identified a nested neural circuit architecture in which a single computation—motion-opponent suppression—is implemented at every processing level across the network. Interestingly, most cells in the lobula plate that are postsynaptic to T4/T5 cells also receive input from LPi cells^14,27^, suggesting that multilevel opponency is not restricted to LPTC circuits, but a general connectivity principle in the lobula plate.

### Conductance mismatch between inhibitory and excitatory synapses

We found a tenfold to 20-fold difference between the number of excitatory and inhibitory synaptic inputs onto VS and LPi cells (Fig. 3 and Extended Data Fig. 3), in line with a recent study^14^. Whereas the physiological impact of synapses of the same type grows linearly with synapse number^42^, our measurements argue that the unitary synaptic conductance of inhibitory synapses in the lobula plate circuit is at least an order of magnitude larger than that of excitatory synapses, thereby compensating for the lower number of synaptic inputs (Fig. 3 and Extended Data Fig. 3). The majority of synaptic connections in the larval and adult fly brain have low synapse numbers^26,43^. Our results show that such anatomically sparse connections—especially when inhibitory and acting on GluClα receptors—can have a functionally strong and important impact^44,45^. This is reminiscent of the mouse visual cortex, where inhibitory neurons only provide around 10% of input synapses to pyramidal cells^46^, yet inhibitory conductances dominate sensory responses^47^. Thus, our study highlights that the relative conductances of different synapse and receptor types are critical when inferring physiological connection strength from anatomical synapse counts.

### Signal rectification by voltage-to-calcium transformation

In contrast to voltage responses, calcium signals only showed weak changes on ND stimulation in all cell types, resulting in small MOIs and arguing for a nonlinear rectifying transformation from voltage to calcium (Extended Data Fig. 2). A similar nonlinear voltage-to-calcium transformation is thought to underlie the emergence of ON and OFF selectivity in *Drosophila* medullary neurons^48^. Mechanistically, such rectifying transformations could plausibly be implemented by the nonlinear gating of voltage-gated calcium channels^49^. Why is the information about opposing motion largely discarded at the neurons’ output stage? For the output signal of a neuron to be motion-opponent, the cell would have to release neurotransmitter at rest and increase or decrease transmitter release depending on the direction of stimulus motion, respectively. This would be energetically expensive and it would lead to increased synaptic conductance and thus reduced response gain in postsynaptic cells. Thus, integrating information about opponent motion directions at the level of the membrane potential and then rectifying the signal before transmitting it to postsynaptic partners is probably beneficial from an energetic and an information theoretical point of view.

### Functional specialization of postsynaptic computations

Why is the lobula plate network implementing a seemingly redundant operation at multiple consecutive processing stages? In addition to biophysical considerations, one possible benefit of such an architecture is that it allows each level to functionally specialize independently and thereby fine-tune the properties of the opponent computation. This way, distinct network layers could implement slightly different arithmetic operations that lead to opponent suppression, despite being implemented by the same cell type. As one example, T4 cells rest at more negative membrane potentials than VS cells and are therefore closer to the reversal potential of chloride^45,50^. Consequently, opponent inhibition might be mainly subtractive, that is, linear, in LPi and VS cells, and more divisive in T4/T5 cells. This would also explain why hyperpolarizing responses to ND motion are much smaller in T4/T5 cells than LPi or VS cells (Fig. 1), despite showing similar amounts of opponent suppression (Fig. 5).

### Function of opponency in relation to the visual environment

Our experiments revealed a gradient of increasing spatiotemporal integration from earlier to later stages of the lobula plate network (Fig. 6). This is similar to the mouse visual system where both receptive field size and temporal integration increase along the anatomical hierarchy of visual areas^51^. We reasoned that the functional roles of the distinct levels of opponency are linked to their different spatial integration scales, which might be reflected in different features of the visual environment across scales. Accordingly, T4/T5 cells with small receptive fields cancel out locally opponent signals that arise by local luminance fluctuations^32^. The intermediate, LPi, level then suppresses uncorrelated image motion over intermediate spatial scales. Importantly, the statistical structure of moving natural scenes alone already leads to the activation of oppositely tuned motion detectors at small spatial scales^32,52^ and might thus be the dominant source of locally opponent motion signals. In general, everything that induces the activation of oppositely tuned T4/T5 cells within the spatial scale of an opponent level will be suppressed by opponent inhibition. In addition to visual features, this might be caused by intrinsic neuronal noise, if correlated across neurons.

At the output layer of the lobula plate network, VS cells can distinguish between different flow fields by integrating motion information over a large visual field in which excitatory signals from the matching part of the receptive field are canceled by inhibitory signals from the nonmatching part^12^. Thereby, the motion-opponent stage at the level of VS cell dendrites acts as a selectivity filter that is matched to particular flow fields encountered during ego motion^12,17,18^. Notably, LPTCs are not the last stage where motion-opponent signals are integrated. In a neural circuit for looming detection, for example, contralateral suppression between visual glomeruli of the two brain hemispheres was found^53^. Additionally, recent studies showed that comparing the activities of oppositely tuned motion-sensitive neurons across the two hemispheres adds another layer of opponency that further enhances selectivity to particular optic flow patterns^54,55^.

### Sublinearity of synaptic integration limits dynamic range

An important functional role of multilevel opponency is linked to the biophysical properties of synaptic integration (Fig. 7). In a perfectly linear system, it is mathematically equivalent if a subtractive operation is carried out in multiple steps or all at once. The interaction of synaptic inputs, however, is intrinsically sublinear and, as a result, the gain of a neuron decreases with increasing total synaptic conductance, thereby limiting its dynamic range^41^. Thus, cancellation of opposing motion at a single neuronal instance can lead to a conductance overload that drives the cell into a low-gain regime. Distributing this computation over several levels allows each level to operate in a range where the transfer function from synaptic conductance to membrane potential is steep and thus bandwidth and information transfer is high, particularly in the presence of noise. Thus, integration of opponently tuned excitation and inhibition at multiple consecutive network layers allows the circuit to achieve high selectivity at the output without compromising sensitivity at every stage of the network. Thus, our study illustrates that the architecture of biological neural networks, in contrast to artificial networks, is not only shaped by the computational operations it must fulfill, but also by the intrinsic biophysical properties of its neuronal constituents.

## Methods

### Fly husbandry

All flies (*Drosophila melanogaster*) were generally raised on standard cornmeal agar medium at 25 °C and 60% humidity on a 12 h light–12 h dark cycle throughout development. For the optogenetic experiments, freshly eclosed flies were transferred to food that was supplemented with yeast paste containing 1 mM all-trans-retinal powder (catalog no. R2500, Sigma-Aldrich). We used female flies for all experiments. For electrophysiology, we used flies that were 1–2 days old. For the calcium or voltage imaging experiments, flies where imaged at an age between 4 and 7 days. Experimental flies that carried RNAi or TeNT transgenes, as well as their respective controls, were transferred to 28.5 °C after eclosion to enhance the strength of transgene expression. A list of all fly genotypes is provided in Supplementary Table 1.

### Electrophysiology

Electrophysiological whole-cell patch clamp recordings were done as described previously^57^. Briefly, flies were waxed to a plexiglass holder with beeswax and the head inserted into an opening in aluminum foil that was mounted on a recording chamber. External saline was added to the preparation, a part of the cuticle on the posterior side of the head was removed with a fine needle and the muscle covering the cell bodies of LPTCs was severed. The glial sheath on the surface of the brain was locally digested by applying Collagenase IV (Thermo Fisher Scientific) through a pipette with an approximate 5-µm opening. When the somata of LPTCs were exposed, whole-cell recordings were performed with patch electrodes (TW150F-4, WPI) pulled to a resistance of 5–9MΩ. For recordings from VS cells that expressed ArcLight, signals were amplified with a BA-1S amplifier (NPI Electronics), low-pass-filtered with a cutoff frequency of 3 kHz and digitized at 10 kHz. For conductance measurements, a MultiClamp 700B (Molecular Devices) amplifier was used. To calculate total conductance changes from current clamp measurements, constant hyperpolarizing holding currents ranging from −200 to 0 pA in steps of 50 pA were injected throughout the stimulus sweeps. Data acquisition was performed with MATLAB v.R2011b (MathWorks) and data analysis was done with MATLAB v.R2013b and Python v.2.7.15.

Normal external saline contained the following: 103 mM NaCl, 3 mM KCl, 5 mM TES, 10 mM trehalose, 10 mM glucose, 3 mM sucrose, 26 mM NaHCO_3_, 1 mM NaH_2_PO_4_, 1.5 mM CaCl_2_ and 4 mM MgCl_2_. Zero Ca^2+^ and high Mg^2+^ external saline was used to silence synaptic transmission and contained: 66 mM NaCl, 3 mM KCl, 5 mM TES, 1 mM NaH_2_PO_4_, 20 mM MgCl_2_, 22 mM Na gluconate, 10 mM trehalose, 5 mM glucose and 26 mM NaHCO_3_. The pH of external solutions was 7.3–7.35 and the osmolarity was around 285 mOsmol kg^−1^. External saline was oxygenated with 95% O_2_ and 5% CO_2_. The internal solution contained the following: 140 mM K-aspartate, 10 mM HEPES, 4 mM Mg-ATP, 0.5 mM Na guanosine 5′-triphosphate, 1 mM EGTA, 1 mM KCl and 0.2 mM Alexa Fluor 568 hydrazide. The pH of the internal solution was adjusted to 7.26 and the osmolarity to approximately 265 mOsmol kg^−1^. The cell bodies of VS and HS cells were targeted for recordings based on their typical anatomical location or guided by the expression of the ArcLight voltage indicator. Additionally, cell types were identified based on the typical response profiles of VS and HS cells to moving gratings and, in most cases, anatomically when cells were properly filled with the Alexa Fluor 568 dye.

### Functional two-photon imaging

For functional voltage or calcium imaging, we used a custom-built two-photon laser scanning microscope that was described previously^11^. Fly cuticle dissections were performed identically as for the electrophysiological experiments and imaged in the same extracellular saline. Images were acquired at a resolution of 64 × 64 or 128 × 64 pixels and a frame rate of 12.6 Hz. Data acquisition was performed in MATLAB v.R2013b using ScanImage v.3.8. Data analysis was performed in Python v.2.7.15 and Python v.3.8.8.

### Immunohistochemistry

Brains from female flies were dissected in cold PBS and fixed in 4% paraformaldehyde (in PBS with 0.1% Triton). Brains were washed three times in PBT (PBS + 0.3% Triton X-100), blocked in 10% normal goat serum (NGS) in PBT and incubated in the primary antibody solution (antibody in 5% NGS in PBT) for 36–48 h. Afterwards, brains were washed in PBT overnight and then incubated in the secondary antibody solution for 48–72 h. Brains were then washed in PBT overnight, briefly washed in PBS and mounted in VECTASHIELD medium (Vector Laboratories). Primary antibodies were used at the following dilutions: anti-nc82 (1:25), anti-GFP (1:1,000) anti-DsRed (1:1,000). We used all secondary antibodies at a dilution of 1:500.

### Confocal microscopy

Images were acquired with the Leica Application Suite X on a Leica SP8 confocal microscope equipped with 488-, 561- and 633-nm lasers and HyD detectors with a Leica 20× or 63× glycerol immersion objective at a resolution of 1,024 × 1,024 pixels. Image processing was performed with ImageJ/Fiji v.1.52e.

### Optogenetic stimulation

Optogenetic stimulation of ChR2-expressing cells was performed as described previously^13^. While obtaining electrophysiological recordings from VS cells, wide-field light pulses were delivered through a 40× (0.8 numerical aperture) water-immersion objective (LUMPlan, Olympus) to the fly brain. A Lambda DG-4 Plus wavelength switcher (Sutter) with a 300 W Xenon Arc lamp served as a light source. ChR2 was excited for 1 s with blue light (472/30 nm) at 3–4 mW mm^−^^2^ as measured under the objective in air.

### Visual stimulation

For the electrophysiological recordings in Extended Data Fig. 1, a custom-built LED arena was used for visual stimulation that was based on a system described previously^58^. The arena spanned 170° in azimuth and 90° in elevation, allowed refresh rates up to 600 Hz and had a maximum luminance of 80 cd m^−^^2^. For functional voltage and calcium imaging and all other electrophysiological recordings, we used a projector-based arena^59^. Visual stimuli were projected onto the back of an opaque cylindrical screen with two micro-projectors (TI DLP LightCrafter 3000). The arena covered 180° in azimuth and 105° in elevation. Visual stimuli were displayed with a refresh rate of 180 Hz and a maximum luminance of 276 ± 48 cd m^−^^2^. All visual stimuli were presented in a randomized manner.

Moving sine-wave gratings (Figs. 1d–f, 2 and 4e–g and Extended Data Figs. 1, 2a–d and 6b–e) were displayed at full contrast, had a wavelength of 30° and moved at 30° per second for 1 s (which corresponds to a temporal frequency of 1 Hz) in eight different directions. Moving bright (ON) and dark (OFF) edges (Extended Data Fig. 6j–m) had a Michelson contrast of 92% and moved at a velocity of 30° per second.

For all TM stimuli (Figs. 5 and 7c–h, Extended Data Figs. 5 and 8a–c and Supplementary Videos 1 and 2), 300 dots with two-dimensional Gaussian profiles (*σ* = 4°) were randomly placed on the arena. For PD or ND motion, 50% of the dots moved in PD or ND while the other 50% of dots remained stationary. For local TM, 50% of dots moved in PD and 50% in ND, respectively. To measure tuning curves (Fig. 5a,b) fractions of moving dots were as follows: PD/ND = 0/0.5, 0.125/0.5, 0.25/0.5, 0.375/0.5, 0.5/0.5, 0.5/0.375, 0.5/0.25, 0.5/0.125 and 0.5/0. The remaining dots were stationary. Coherence was defined as ((fraction of dots moving in PD) − (fraction of dots moving in ND)) × 2. Dots moved at a velocity of 30° per second and had a maximal luminance value of 250 on a background with luminance 10, resulting in a Michelson contrast of 92%. Global TM stimuli had identical parameters for dot size and dot number, but dots moved coherently in PD or ND in the upper or lower half of the screen. To ensure that VS cells were stimulated with equal amounts of PD and ND motion across their receptive fields on average, we presented two variations of each stimulus in which we switched the parts of the screen (upper half or lower half) on which PD or ND motion was shown. The data of these two stimulus variations were then averaged to calculate membrane potential or conductance changes. Stimulus duration was 8 s for the calcium imaging experiments and 5 s for electrophysiology.

Dot motion stimuli (Fig. 1g,k and Extended Data Figs. 2e,j and 6f–i) were identical to TM stimuli, with the exception that all dots moved coherently in the same direction over the entire extent of the visual arena. Dot motion stimuli were used instead of sine-wave gratings to measure directional tuning curves in T4 cells with the voltage indicator (Fig. 1g,k) because T4 cells respond to ON and OFF components of the grating with the opposite sign^60^, which can lead to response reduction or cancellation when averaging multiple cells. As a control, we thus ensured that the calcium responses of T4 cells were highly similar when comparing sine-wave grating and dot motion stimulation (Extended Data Fig. 2d,e).

The stochastic motion stimulus (Fig. 6a–e) consisted of 25 randomly distributed circular windows with sizes of 10°. Inside each window, a sine-wave grating with 100% contrast and a wavelength of 20° moved at 1 Hz for 1 s in the PD of the cell type under investigation. The positions of the circular windows were changed randomly every second and the stimulus was displayed for 720 s in total. Reverse correlation of the cells’ response traces with the stimulus then yielded motion-sensitive spatiotemporal receptive fields. The spatial components of the receptive fields were fitted with a one-dimensional Gaussian profile to determine the receptive field sizes in azimuth and elevation.

The annulus motion stimulus (Fig. 6f–h) consisted of a circular region with a 15° diameter in which a sine-wave grating with full contrast and a wavelength of 30° was moving with 1 Hz in the PD of the respective T4/T5 cell population that was imaged. This inner circle was surrounded by a 5° wide gray region (50% contrast), which again was surrounded by an annulus with a 15° width. In the annulus region, a second sine-wave grating with a 30° wavelength and full contrast was moving in one of eight different directions at 1 Hz temporal frequency. The stimulus was centered on the receptive field of a T4/T5 cell by first selecting an axonal region and then moving the stimulus manually across the screen until the axon terminal responded maximally. Both gratings in the center and annulus moved either individually or simultaneously for 3 s.

### Connectomic analysis

To analyze the *Drosophila* hemibrain connectomic data, we used custom-written Python code to query synaptic connections between anatomically identified neuron reconstructions from neuprint (http://neuprint.janelia.org)^61^. Neuronal types were identified based on arborizations of cell types in respective lobula plate layers and by visually matching morphology from EM reconstructions to light microscopy data (see also the Results section).

### Calculation of estimated synaptic conductance ratios

Calculations for estimating the synaptic conductance ratio (Extended Data Fig. 3b) were done as follows. The steady-state change in postsynaptic voltage (Δ*V*_syn_) in response to synaptic input is given by^41^:

$$\Delta {V}_{{\mathrm {syn}}}=\frac{{n}_{{\mathrm {syn}}}\times {g}_{{\mathrm {syn}}}\times {E}_{{\mathrm {syn}}}}{{n}_{{\mathrm {syn}}}\times {g}_{{\mathrm {syn}}}+{g}_{{\mathrm {leak}}}}$$

With *n*_syn_ denoting the number of synapses, *g*_syn_ the conductance of a single synapse, *E*_syn_ the synaptic reversal potential relative to the resting membrane potential (i.e. the synaptic driving force) and *g*_leak_ the leak conductance. Setting voltage changes caused by inhibitory and excitatory input equal ($\varDelta {V}_{\mathrm{e}}=-\varDelta {V}_{\mathrm{i}}$) allows the expression of the synaptic conductance ratio $\left(\frac{{g}_{{\mathrm{i}}}}{{g}_{{\mathrm {e}}}}\right)$ as:

$$\frac{{g}_{{\mathrm {i}}}}{{g}_{{\mathrm {e}}}}=\frac{{g}_{{{\mathrm {leak}}}}}{\left(\frac{{n}_{{\mathrm {e}}}}{{n}_{{\mathrm {i}}}}\right)\times \left({n}_{{\mathrm {i}}}\times {g}_{{\mathrm {i}}}-\frac{{E}_{{\mathrm {e}}}}{{E}_{{\mathrm {i}}}}\times {g}_{{{\mathrm {leak}}}}\right)}$$

This gives:

$$\,\frac{{g}_{{\mathrm {i}}}}{{g}_{{\mathrm {e}}}}=\left(\frac{{n}_{{\mathrm {e}}}}{{n}_{{\mathrm {i}}}}\right)\times \left(\frac{{n}_{{\mathrm {i}}}\times {g}_{{\mathrm {i}}}}{{g}_{{{\mathrm {leak}}}}}-\frac{{E}_{{\mathrm {e}}}}{{E}_{{\mathrm {i}}}}\right)$$

The parameters used for the calculations were $\frac{{n}_{{\mathrm {e}}}}{{n}_{{\mathrm {i}}}}$ = 8.64 (VS cells), 18.91 (LPi3-4 cells) and 17.59 (LPi4-3 cells) as obtained from the connectomic analysis (Fig. 3h,i), and ${E}_{{\mathrm {e}}}=40\,{\mathrm {mV}}$ and ${E}_{{\mathrm {i}}}=-20\,{\mathrm {mV}}$ based on the electrophysiological measurements of the synaptic reversal potentials for cholinergic and glutamatergic synapses^45^.

### Computational modeling of the lobula plate network

The model was updated at a temporal resolution of 10 ms. T4/T5 cell responses were modeled based on an earlier study^35^. Briefly, the output of a motion detector with three spatially separated input lines (A, B, C) was calculated: visual input to lines A and C was low-pass-filtered with *τ* = 50 ms whereas the input to line B was high-pass-filtered with *τ* = 250 ms. The output of the detector was then calculated according to (A × B/(C + DC)) with DC = 0.02 followed by rectification.

The first stage of the network was modeled as an array of 30 × 28 T4/T5 elementary motion detectors. The output of this stage converged onto an array of 6 × 6 LPi cells. At the final stage, a single VS cell integrated input from T4/T5 and LPi cells over the whole spatial extent of the stimulus. Synaptic inputs to all cell types, with the exception of VS cells, were integrated linearly. VS cell responses were calculated with a conductance-based model. The reversal potentials of excitatory and inhibitory synapses were −20 mV and +40 mV with respect to the resting potential. The gains of synaptic connections were as follows: T4/T5-LPi, 1.0; T4/T5-VS, 1.0; LPi-VS, 1.0; LPi-LPi, 1.0; and LPi-T4/T5, 0.5. Synapses were rectified with a threshold at 0. The time constants of all neurons were set to 20 ms.

### Visual stimuli for modeling

Visual stimuli had a spatial extent of 180 × 180 pixels, where each pixel corresponded to 1° of visual space. Visual stimuli were similar to those used in the experiments. Sine-wave gratings had a spatial wavelength of 30° and moved at a temporal frequency of 1 Hz. For TM stimuli, 200 Gaussian-shaped dots (*σ* = 5 pixels) were randomly placed on the stimulation area. Dots moved at a velocity of 100° per second. Random placement of dots for TM stimulation resulted in slightly variable model output. Therefore, simulations were run 300 times and the model outputs of each run were averaged. This also allowed for a statistical comparison of responses to different stimuli. The computational model was implemented in Python v.3.8.8.

### Analysis of functional imaging data

Preprocessing of functional imaging data was performed as described in an earlier publication^59^. Image stacks were first automatically registered using cross-correlation of pixel intensity values to correct for brain movements in the *xy* plane. ROIs were then manually selected based on the average image projected over all time frames. To determine spatiotemporal receptive fields (Fig. 6a–e), we selected ROIs that corresponded to individual axons for VS cells and single axonal boutons for LPi and T4/T5 cells. For all other experiments, we selected ROIs that corresponded to multiple individual cells because individual cells or subcellular structures responded similarly to the respective stimuli. Relative fluorescence changes (Δ*F/F*) were obtained by using an automatic baseline detection algorithm. Raw data were first smoothed with a Gaussian filter (full width at half maximum = 1 s). Minima within a 10-s-long sliding window were then extracted and the resulting trace smoothed with another Gaussian filter. The resulting dynamic baseline was then set as *F*_0_ from which relative fluorescence changes were computed according to Δ*F/F* = (*F* − *F*_0_)/*F*_0_.

Individual stimuli were presented four to ten times in randomized order. The average Δ*F/F* value 1 s before the stimulus start was used as the baseline. Responses to each individual stimulus trial were then averaged. Mean responses were calculated by averaging data points over the visual stimulation period for voltage imaging. For calcium imaging, because of the slow dynamics of the calcium indicator, we averaged data points from the stimulus start to including 1 s after the stimulus end. Some response traces from the voltage imaging experiments (Fig. 1g and Extended Data Fig. 5g) and electrophysiology (Fig. 7c–f) were smoothed with a first-order Savitzky–Golay filter (windows = 250 ms (voltage imaging), 70 ms (voltage responses for electrophysiology) and 150 ms (conductances for electrophysiology), for display purposes.

### Analysis of electrophysiological data

For the electrophysiological data, to account for slow potential drift, baseline correction was performed by subtracting the average membrane potential calculated over 1 s before the stimulus start from the response trace. Responses to individual stimulus trials were then averaged and mean responses calculated by averaging over the entire stimulation period. Total conductance changes in response to visual stimulation were determined by calculating the inverse of the slope of a linear regression of voltage responses on different holding currents (ranging from −200 to 0 pA in steps of 50 pA).

### Calculation of response indices

L_Dir_ indices were calculated as the length of the normalized response vector according to:

$${{\rm{L}}}_{{\rm{Dir}}}=\left|\frac{{\sum }_{{{\varphi }}}\vec{r}({{\varphi }})}{{\sum }_{{{\varphi }}}\left|\vec{r}({{\varphi }})\right|}\right|$$

where $\vec{r}({{\varphi }})$ is a vector with the stimulus direction *φ* as vector angle and the corresponding neuronal response as vector length. The PD of a response then corresponded to the angle of this normalized response vector.

MOIs were calculated as the ratio between the response vector magnitude of the depolarizing responses $\left|\vec{{R}_{{\mathrm{dep}}}}\right|=\left|{\sum }_{\varphi =1}^{n}\vec{{r}_{{\mathrm{dep}}(\varphi )}}\right|$ and the response vector magnitude of the hyperpolarizing responses $\left|\vec{{R}_{\mathrm{h{yp}}}}\right|=\left|{\sum }_{\varphi =1}^{n}\vec{{r}_{\mathrm{h{yp}}(\varphi )}}\right|$ according to:

$${{\mathrm {for}}}\,\left|\vec{{R}_{\mathrm{h{yp}}}}\right| > \left|\vec{{R}_{{\mathrm{dep}}}}\right|:{\mathrm{{MOI}}}=\cos \theta \frac{\left|\vec{{R}_{{\mathrm{dep}}}}\right|}{\left|\vec{{R}_{\mathrm{h{yp}}}}\right|}$$

$${\mathrm {for}}\,\left|\vec{{R}_{{\mathrm{hyp}}}}\right| < \left|\vec{{R}_{{\mathrm{dep}}}}\right|:{\mathrm{{MOI}}}=\cos \theta \frac{\left|\vec{{R}_{{\mathrm{hyp}}}}\right|}{\left|\vec{{R}_{{\mathrm{dep}}}}\right|}$$

Motion-opponent suppression was defined as: $\frac{\left|{R}_{{\mathrm{PD}}}\right|-\left|{R}_{{\mathrm{TM}}}\right|}{\left|{R}_{{\mathrm{PD}}}\right|}$, where *R* denotes the mean response magnitude to PD or TM stimulation.

To calculate the PDs of suppression for the annulus motion stimulus experiments, we first calculated a response suppression ratio, which was defined as: $1-\frac{{R}_{({\mathrm{center}+\mathrm{{annulus}}})}}{{R}_{(\mathrm{{center})}}+{R}_{(\mathrm{{annulus}})}}$

Using this metric, we then calculated a normalized response suppression vector in an equivalent manner as for determining the L_Dir_ index. The direction of this vector then indicates the PD of suppression.

### Statistics and reproducibility

No statistical methods were used to predetermine sample sizes, but our sample sizes are similar to those reported in previous publications^12,20,27^. Data collection and analysis were not performed blind to the conditions of the experiments. However, data acquisition and analysis were performed identically for all genotypes. Only animals that did not show any detectable changes in membrane potential or fluorescence were excluded from the analysis because this is indicative of poor health after microsurgery. All immunostaining (Fig. 4a–d and Extended Data Fig. 4) were repeated at least three times with similar results.

### Statistical analysis

To test for the normality of data distributions, we first performed a Shapiro–Wilk test followed by a Levene test to assess the equality of variances. Based on the outcome of these tests, we then performed either a Student’s *t*-test, Welch’s *t*-test or Mann–Whitney *U*-test. Statistical significance for paired data with non-normal distribution was assessed with a Wilcoxon signed-rank test. Linear correlation was assessed with a Wald test. To test whether the motion-opponent suppression was directionally tuned (Fig. 6h), we applied a Rayleigh *z*-test. Holm’s post-hoc correction was applied when more than two experimental groups were compared. NS, *P* > 0.05; *P < 0.05, **P < 0.01, ***P < 0.001. Statistical analysis was performed in Python v.2.7.15 or Python v.3.8.8 using scipy v.1.6.2, statsmodels v.0.12.2, scikit_posthocs v.0.6.7 and astropy v.2.0.6 packages. More details on statistical analysis and exact *P* values are provided in Supplementary Table 2.

### Availability of materials

This study did not generate new unique reagents. The sources of the experimental animals, reagents and software are listed in Supplementary Table 3.

### Reporting summary

Further information on research design is available in the Nature Portfolio Reporting Summary linked to this article.

## Online content

Any methods, additional references, Nature Portfolio reporting summaries, source data, extended data, supplementary information, acknowledgements, peer review information; details of author contributions and competing interests; and statements of data and code availability are available at 10.1038/s41593-023-01443-z.

### Supplementary information

Supplementary Information Supplementary Tables 1–3.

Reporting Summary

Supplementary Video 1 Local transparent motion stimulus.

Supplementary Video 2 Global transparent motion stimulus.

### Source data

Source Data Fig. 1 Source data.

Source Data Fig. 2 Source data.

Source Data Fig. 3 Source data.

Source Data Fig. 4 Source data and image files.

Source Data Fig. 5 Source data.

Source Data Fig. 6 Source data.

Source Data Fig. 7 Source data.

Source Data Extended Data Fig. 1 Source data.

Source Data Extended Data Fig. 2 Source data.

Source Data Extended Data Fig. 3 Source data.

Source Data Extended Data Fig. 4 Image files.

Source Data Extended Data Fig. 5 Source data.

Source Data Extended Data Fig. 6 Source data.

Source Data Extended Data Fig. 7 Source data.

Source Data Extended Data Fig. 8 Source data.

## Data Availability

All data generated in this paper are publicly available at https://gin.g-node.org/gammer/Ammer_et_al_2023.git (ref. ^56^). The connectomic data were published by the Janelia Research Campus^26^ and are available at https://neuprint.janelia.org. Source data are provided with this paper.

## Code availability

All code used for the analysis is publicly available at https://gin.g-node.org/gammer/Ammer_et_al_2023.git (ref. ^56^).
