# Supplementary material for: Multilevel visual motion opponency in Drosophila
Source: Nat Neurosci. 2023 Oct 2;26(11):1894–905. doi: 10.1038/s41593-023-01443-z (PMC10620086; doi:10.1038/s41593-023-01443-z)
Supplement: Supplementary file 1 — Supplementary Tables 1–3. [file 41593_2023_1443_MOESM1_ESM.pdf]

nature neuroscience

Article

https://doi.org/10.1038/s41593-023-01443-z

Multilevel visual motion opponency in *Drosophila*

In the format provided by the
authors and unedited

nature neuroscience

Article https://doi.org/10.1038/s41593-023-01443-z

Multilevel visual motion opponency in *Drosophila*

In the format provided by the
authors and unedited

Supplementary Table 1. List of all experimental genotypes

Figure	Short Name	Genotype
**Figure 1**b-c	T4/T5 > ChR2	norpA^{7}; UAS-ChR2-H134R; R42F06-Gal4
	VS > ChR2	w+; UAS-ChR2-H134R; R24E09-Gal4
**Figure 1**d,h	VS > ArcLight	w+; UAS-ArcLight/ +; R24E09-Gal4/ +
**Figure 1**e,i	LPi3-4 > ArcLight	w+; UAS-ArcLight/ +; R20D01-Gal4/ +
**Figure 1**f,j	LPi4-3 > ArcLight	w+; UAS-ArcLight/ +; R38G02-Gal4/ +
**Figure 1**g,k	T4c > ArcLight	w+/ w-; VT15785-AD/ UAS-ArcLight; VT50384-DBD/ +
**Figure 2**a-c	VS > ArcLight (control)	w+/ w-; UAS-ArcLight/ +; R24E09-Gal4/ lexAop-TNT
	VS > ArcLight (T4/T5c block)	w+/ w-; UAS-ArcLight/ +; R24E09-Gal4/ VT50384-lexA, lexAop-TNT
**Figure 2**d-f	LPi3-4 > ArcLight (control)	w+/ w-; UAS-ArcLight/ +; R20D01-Gal4/ lexAop-TNT
	LPi3-4 > ArcLight (T4/T5c block)	w+/ w-; UAS-ArcLight/ +; R20D01-Gal4/ VT50384-lexA, lexAop-TNT
**Figure 2**g-i	LPi4-3 > ArcLight (control)	w+/ w-; UAS-ArcLight/ +; R38G02-Gal4/ lexAop-TNT
	LPi4-3 > ArcLight (T4/T5c block)	w+/ w-; UAS-ArcLight/ +; R38G02-Gal4/ VT50384-lexA, lexAop-TNT
**Figure 3**i-j	wildtype	Canton S
**Figure 4**a	VS > GluClα-GFP	w-; VT58487-AD/ UAS-GluClα-GFP; R35G07-DBD/ UAS-tdTomato
**Figure 4**b	LPi3-4 > GluClα-GFP	w-; VT61221-AD/ UAS-GluClα-GFP; VT45575-DBD/ UAS-tdTomato
**Figure 4**c	LPi4-3 > GluClα-GFP	w-; R38G02-AD/ UAS-GluClα-GFP; R24A07-DBD/ UAS-tdTomato
**Figure 4**d	T4/T5c > GluClα-GFP	w-; UAS-GluClα-GFP/+; VT50384-Gal4/ UAS-tdTomato
**Figure 4**e-g	LPi4-3 > ArcLight (RNAi control)	w+; UAS-ArcLight/ UAS-GluRIA-RNAi; R38G02-Gal4/ +
	LPi4-3 > ArcLight (GluClα RNAi)	w+; UAS-ArcLight/ UAS-GluClα-RNAi; R38G02-Gal4/ +
**Figure 5**a-b	TNT control	w+/ w-; +; lexAop-TNT/ +
	T4/T5c block	w+/ w-; +; VT50384-lexA, lexAop-TNT/ +
**Figure 5**c-d	LPi4-3 > GC6f (control)	w+/ w-; UAS-GCaMP6f/ +; R38G02-Gal4/ lexAop-TNT
	LPi4-3 > GC6f (T4/T5c block)	w+/ w-; UAS-GCaMP6f/ +; R38G02-Gal4/ VT50384-lexA, lexAop-TNT
**Figure 5**e-f	T4/T5c > GC6m (control)	w+/ w-; UAS-TNT/ +; VT50384-lexA, lexAop-GCaMP6m/ +
	T4/T5c > GC6m (LPi4-3 block)	w+/ w-; UAS-TNT/ R38G02-AD; VT50384-lexA, lexAop-GCaMP6m/ R24A07-DBD
**Figure 6**a-e	VS > GC6f	w+/ w-; UAS-GCaMP6f/ +; UAS-GCaMP6f/ R24E09-Gal4
	LPi3-4 > GC6f	w+/ w-; UAS-GCaMP6f/ +; UAS-GCaMP6f/ R20D01-Gal4
	LPi4-3 > GC6f	w+/ w-; UAS-GCaMP6f/ +; UAS-GCaMP6f/ R38G02-Gal4
	T4/T5c > GC6f	w+/ w-; UAS-GCaMP6f/ +; UAS-GCaMP6f/ VT50384-Gal4
**Figure 6**g-h	T4/T5c > GC6f	w+/ w-; UAS-GCaMP6f/ +; UAS-GCaMP6f/ VT50384-Gal4
	T4/T5 > GC6f (control)	w+/ w-; UAS-GCaMP6f/ +; R42F06-Gal4/ +
	T4/T5 > GC6f (T4/T5c block)	w+/ w-; UAS-GCaMP6f/ +; R42F06-Gal4/ VT50384-lexA, lexAop-TNT
**Figure 7**c-h	wildtype	Canton S
**Ext. Fig. 1**	VS > ArcLight	w+; UAS-ArcLight/ +; R24E09-Gal4/ +
**Ext. Fig. 2**	VS > GC6f	w+/ w-; UAS-GCaMP6f/ +; UAS-GCaMP6f/ R24E09-Gal4
	LPi3-4 > GC6f	w+/ w-; UAS-GCaMP6f/ +; UAS-GCaMP6f/ R20D01-Gal4
	LPi4-3 > GC6f	w+/ w-; UAS-GCaMP6f/ +; UAS-GCaMP6f/ R38G02-Gal4
	T4c > GC6f	w+; VT15785-AD/ UAS-GCaMP6f; VT50384-DBD/ UAS-GCaMP6f
**Ext. Fig. 3**c	wildtype	Canton S
**Ext. Fig. 4**a	LPi3-4 > nAChRα7-GFP	w-; VT61221-AD/ UAS-tdTomato; VT45575-DBD/ UAS-nAChRα7-GFP
**Ext. Fig. 4**b	LPi4-3 > nAChRα7-GFP	w-; R38G02-AD/ UAS-tdTomato; R24A07-DBD/ UAS-nAChRα7-GFP
**Ext. Fig. 5**a,b	LPi4-3 > GC6f	w+/ w-; UAS-GCaMP6f/ +; UAS-GCaMP6f/ R38G02-Gal4
**Ext. Fig. 5**c,d	T4/T5c > GC6f	w+; UAS-GCaMP6f/+; VT50384-Gal4/+
**Ext. Fig. 5**e,f	T4c > GC6f	w+; VT15785-AD/ UAS-GCaMP6f; VT50384-DBD/ UAS-GCaMP6f
**Ext. Fig. 5**g,h	T4c > ArcLight	w+/ w-; VT15785-AD/ UAS-ArcLight; VT50384-DBD/ +
**Ext. Fig. 6**	T4c > GC6f	w+; VT15785-AD/ UAS-GCaMP6f; VT50384-DBD/ UAS-GCaMP6f
**Ext. Fig. 8**a-c	wildtype	Canton S

Supplementary Table 2. Statistical analysis

Figure	Comparison	Statistical test	p-value	
**Figure 1**l	Correlation: LPi3-4 – LPi4-3	Wald test	$1.1407*10^{-8}$	***
**Figure 2**c	MOI: VS (ctrl) – VS (T4/T5c block)	Welch's t-test	$8.1344*10^{-4}$	***
	LDir: VS (ctrl) – VS (T4/T5c block)	Welch's t-test	$5.3057*10^{-4}$	***
**Figure 2**f	MOI: LPi3-4 (ctrl) – LPi3-4 (T4/T5c block)	Welch's t-test	$1.2751*10^{-3}$	**
	LDir: LPi3-4 (ctrl) – LPi3-4 (T4/T5c block)	Welch's t-test	$3.1044*10^{-3}$	**
**Figure 2**i	MOI: LPi4-3 (ctrl) – LPi4-3 (T4/T5c block)	Welch's t-test	$2.8558*10^{-5}$	***
	LDir: LPi4-3 (ctrl) – LPi4-3 (T4/T5c block)	Welch's t-test	$6.5523*10^{-3}$	**
**Figure 3**j	PD local – ND local	Wilcoxon signed-rank test	0.32587	ns
**Figure 4**g	MOI: LPi4-3 (ctrl) – LPi4-3 (GluClα-RNAi)	Welch's t-test	$9.5774*10^{-5}$	***
	LDir: LPi4-3 (ctrl) – LPi4-3 (GluClα-RNAi)	Welch's t-test	0.01865	*
**Figure 5**b	MSI: VS (ctrl) – VS (T4/T5c block)	Student's t-test	$1.0934*10^{-5}$	***
**Figure 5**d	PD: LPi4-3 (ctrl) – LPi4-3 (T4/T5c block)	Welch's t-test	0.40606	ns
	ND: LPi4-3 (ctrl) – LPi4-3 (T4/T5c block)	Welch's t-test	$9.0649*10^{-3}$	**
	TM: LPi4-3 (ctrl) – LPi4-3 (T4/T5c block)	Welch's t-test	$4.9512*10^{-4}$	***
	MSI: LPi4-3 (ctrl) – LPi4-3 (T4/T5c block)	Student's t-test	$7.7754*10^{-5}$	***
**Figure 5**f	PD: T4/T5c (ctrl) – T4/T5c (LPi4-3 block)	Welch's t-test	0.82811	ns
	ND: T4/T5c (ctrl) – T4/T5c (LPi4-3 block)	Welch's t-test	0.44474	ns
	TM: T4/T5c (ctrl) – T4/T5c (LPi4-3 block)	Welch's t-test	$1.1206*10^{-3}$	**
	MSI: T4/T5c (ctrl) – T4/T5c (LPi4-3 block)	Student's t-test	$2.5241*10^{-5}$	***
**Figure 6**d	RF area: VS – LPi3-4	Mann-Whitney U-test, Holm-corrected	$2.3722*10^{-16}$	***
	RF area: VS – LPi4-3	Mann-Whitney U-test, Holm-corrected	$3.3756*10^{-16}$	***
	RF area: LPi3-4 – T4/T5c	Mann-Whitney U-test, Holm-corrected	$2.0986*10^{-65}$	***
	RF area: LPi4-3 – T4/T5c	Mann-Whitney U-test, Holm-corrected	$1.5924*10^{-61}$	***
**Figure 6**e	tau: VS – LPi3-4	Mann-Whitney U-test, Holm-corrected	$3.3009*10^{-12}$	***
	tau: VS – LPi4-3	Mann-Whitney U-test, Holm-corrected	$6.4809*10^{-13}$	***
	tau: LPi3-4 – T4/T5c	Mann-Whitney U-test, Holm-corrected	$1.1806*10^{-31}$	***
	tau: LPi4-3 – T4/T5c	Mann-Whitney U-test, Holm-corrected	$7.7733*10^{-18}$	***
**Figure 6**h	T4/T5c	Rayleigh z-test	0.02834	*
	T4/T5d	Rayleigh z-test	0.02287	*
	T4/T5d (T4/T5c block)	Rayleigh z-test	0.66186	ns
**Figure 7**g	Model with inhibition: local – global	paired Student's t-test	$1.3108*10^{-169}$	***
	Model w/o inhibition: local – global	paired Student's t-test	0.53761	ns
	Experiment: local – global	Wilcoxon signed-rank test	0.01738	*
**Figure 7**h	Model with inhibition: PD/ND sum – local	paired Student's t-test	$9.5638*10^{-252}$	***
	Model w/o inhibition: PD/ND sum – local	paired Student's t-test	$6.6408*10^{-175}$	***
	Experiment: PD/ND sum – local	Wilcoxon signed-rank test	0.00228	**
**Figure 7**l	Correlation: Diff. gain – diff. conduct. (PD)	Wald test	$1.3917*10^{-3}$	**
	Correlation: Diff. gain – diff. conduct. (ND)	Wald test	$3.6991*10^{-3}$	**
**Ext. Fig. 1**c	Correlation: VS (ephys) – VS (ArcLight)	Wald test	$6.2240*10^{-7}$	***
**Ext. Fig. 1**d	MOI: VS (ephys) – VS (ArcLight)	Student's t-test	0.22568	ns
	LDir: VS (ephys) – VS (ArcLight)	Student's t-test	0.58362	ns
**Ext. Fig. 2**k	MOI: VS (ArcLight) – VS (GCaMP)	Student's t-test	$1.3514*10^{-7}$	***
	MOI: LPi3-4 (ArcLight) – LPi3-4 (GCaMP)	Student's t-test	$2.9343*10^{-6}$	***
	MOI: LPi4-3 (ArcLight) – LPi4-3 (GCaMP)	Student's t-test	$7.6028*10^{-7}$	***
	MOI: T4c (ArcLight) – T4c (GCaMP)	Student's t-test	0.90496	ns
**Ext. Fig. 2**l	LDir: VS (ArcLight) – VS (GCaMP)	Student's t-test	$2.5110*10^{-4}$	***
	LDir: LPi3-4 (ArcLight) – LPi3-4 (GCaMP)	Student's t-test	$2.9359*10^{-6}$	***
	LDir: LPi4-3 (ArcLight) – LPi4-3 (GCaMP)	Student's t-test	$1.7654*10^{-7}$	***
	LDir: T4c (ArcLight) – T4c (GCaMP)	Student's t-test	0.13371	ns
**Ext. Fig. 2**m	PD: VS (ArcLight) – VS (GCaMP)	Student's t-test	0.50524	ns
	PD: LPi3-4 (ArcLight) – LPi3-4 (GCaMP)	Student's t-test	0.79030	ns
	PD: LPi4-3 (ArcLight) – LPi4-3 (GCaMP)	Student's t-test	0.18735	ns
	PD: T4c (ArcLight) – T4c (GCaMP)	Student's t-test	0.28244	ns
**Ext. Fig. 3**c	PD local – ND local	Wilcoxon signed-rank test	0.32587	ns
	PD global – ND global	Wilcoxon signed-rank test	0.64166	ns
**Ext. Fig. 6d**	PD (90°) sine wave: dend – axon	paired Student's t-test	0.06152	ns
	ND (270°) sine wave: dend – axon	paired Student's t-test	$3.2141*10^{-3}$	**
**Ext. Fig. 6**e	MOI sine wave: dend – axon	paired Student's t-test	$4.0932*10^{-3}$	**
	LDir sine wave: dend – axon	paired Student's t-test	0.01250	*
	PrefDir sine wave: dend – axon	paired Student's t-test	0.05741	ns
**Ext. Fig. 6**h	PD (90°) dot motion: dend – axon	paired Student's t-test	0.82709	ns
	ND (270°) dot motion: dend – axon	paired Student's t-test	$2.7845*10^{-3}$	**
**Ext. Fig. 6**i	MOI dot motion: dend – axon	paired Student's t-test	0.45529	ns

	LDir dot motion: dend – axon	paired Student's t-test	0.14394	ns
	PrefDir dot motion: dend – axon	paired Student's t-test	0.25177	ns
**Ext. Fig. 6**l	PD (90°) edges: dend – axon	Student's t-test	0.37722	ns
	ND (270°) edges: dend – axon	Student's t-test	0.59320	ns
**Ext. Fig. 6**m	MOI edges: dend – axon	Student's t-test	0.45927	ns
	LDir edges: dend – axon	Student's t-test	0.05213	ns
	PrefDir edges: dend – axon	Student's t-test	0.01100	*
**Ext. Fig. 8**c	Model with inhibition: PD/ND sum – global	paired Student's t-test	1.0	ns
	Model w/o inhibition: PD/ND sum – global	paired Student's t-test	1.0	ns
	Experiment: PD/ND sum – global	Wilcoxon signed-rank test	0.46300	ns

Supplementary Table 3. Sources of experimental animals, reagents and software

Resource	Source	Identifier
**Antibodies**		
anti-brp (nc82) mouse monoclonal antibody	DSHB	RRID: AB_2314866
anti-GFP chicken polyclonal antibody	Rockland	Cat# 600-901-215S
anti-DsRed rabbit polyclonal antibody	Takara Bio	RRID: AB_10013483
donkey anti-chicken-Alexa-488	Jackson Immuno Research	Cat# 703-545-155
goat anti-rabbit-Alexa-568	Invitogen	Cat# A-11011
goat anti-mouse-Alexa-647	Invitrogen	Cat# A32728
**Experimental animals**		
w-; +; R20D01-Gal4 (LPi3-4 cells)	BDSC	RRID: BDSC_48889
w-; VT61221-AD; VT45575-DBD (LPi3-4 cells)	BDSC	RRID: BDSC_86823
w-; +; R24E09-Gal4 (VS/HS cells)	BDSC	RRID: BDSC_49083
w-; VT58487-AD; R35G07-DBD (VS/HS cells)	BDSC	RRID: BDSC_86824
w-; +; R38G02-Gal4 (LPi4-3 cells)	BDSC	RRID: BDSC_50016
w-; R38G02-AD; R24A07-DBD (LPi4-3 cells)	BDSC	RRID: BDSC_76004
w-; +; R42F06-Gal4 (T4/T5 cells)	BDSC	RRID: BDSC_41253
w-; VT15785-AD; VT50384-DBD (T4c cells)	Tirian et al., bioRxiv, 2017	N/A
w-; +; VT50384-Gal4/Tm6b (T4/T5c cells)	Tirian et al., bioRxiv, 2017	N/A
w-; +; VT50384-lexA/Tm6b (T4/T5c cells)	Tirian et al., bioRxiv, 2017	N/A
w-; 20xUAS-IVS-GCaMP6f; +	BDSC	RRID: BDSC_42747
w-; +; 20xUAS-IVS-GCaMP6f	BDSC	RRID: BDSC_52869
w-; +; 13xlexAop-IVS-GCaMP6m	BDSC	RRID: BDSC_44277
w-; UAS-ArcLight; +	BDSC	RRID: BDSC_51057
w-; +; UAS-ArcLight	BDSC	RRID: BDSC_51056
w-; UAS-TNT; +	BDSC	RRID: BDSC_28838
w-; +; 13xlexAop-IVS-TNT:HA	Karuppudurai et al., Neuron, 2014	N/A
w+; UAS-GluClα-RNAi; +	BDSC	RRID: BDSC_53356
w+; UAS-GluRIA-RNAi; +	BDSC	RRID: BDSC_40844
w-; UAS-GluClα-GFP; +	BDSC	RRID: BDSC_92151
w-; UAS-Rdl-GFP; +	BDSC	RRID: BDSC_92150
w-; UAS-nAChR-Dα7-GFP; +	Fendl et al., eLife, 2020	N/A
w-; +; UAS-tdTomato	BDSC	RRID: BDSC_32221
norpA^{7}; +; +	BDSC	RRID: BDSC_5685
w-; UAS-ChR2-H134R-mCherry; +	Mauss et al., JNeurosci, 2014	N/A
**Software and algorithms**		
MATLAB	MathWorks	mathworks.com
Python 2.7.15 and 3.8.8	Python Software Foundation	python.org
ScanImage 3.8	Vidrio Technologies	scanimage.vidriotechnologies.com
ImageJ/Fiji	National Institutes of Health	imagej.nih.gov/ij/
neuPrint	Janelia Research Campus	neuprint.janelia.org
**Deposited data**		
Data and code for this paper	G-Node GIN	doi: 10.12751/g-node.7v3pe6
