# Supplementary material for: Multilevel visual motion opponency in Drosophila
Source: Nat Neurosci. 2023 Oct 2;26(11):1894–905. doi: 10.1038/s41593-023-01443-z (PMC10620086; doi:10.1038/s41593-023-01443-z)
Supplement: Supplementary file 2 — Reporting Summary [file 41593_2023_1443_MOESM2_ESM.pdf]

Corresponding author(s): Ammer Georg
Last updated by author(s): Aug 26, 2023

Reporting Summary

Nature Portfolio wishes to improve the reproducibility of the work that we publish. This form provides structure for consistency and transparency in reporting. For further information on Nature Portfolio policies, see our Editorial Policies and the Editorial Policy Checklist.

Statistics

For all statistical analyses, confirm that the following items are present in the figure legend, table legend, main text, or Methods section.

n/a	Confirmed	
☐	☒	The exact sample size (*n*) for each experimental group/condition, given as a discrete number and unit of measurement
☐	☒	A statement on whether measurements were taken from distinct samples or whether the same sample was measured repeatedly
☐	☒	The statistical test(s) used AND whether they are one- or two-sided<br>*Only common tests should be described solely by name; describe more complex techniques in the Methods section.*
☒	☐	A description of all covariates tested
☐	☒	A description of any assumptions or corrections, such as tests of normality and adjustment for multiple comparisons
☐	☒	A full description of the statistical parameters including central tendency (e.g. means) or other basic estimates (e.g. regression coefficient) AND variation (e.g. standard deviation) or associated estimates of uncertainty (e.g. confidence intervals)
☐	☒	For null hypothesis testing, the test statistic (e.g. *F*, *t*, *r*) with confidence intervals, effect sizes, degrees of freedom and *P* value noted<br>*Give P values as exact values whenever suitable.*
☒	☐	For Bayesian analysis, information on the choice of priors and Markov chain Monte Carlo settings
☒	☐	For hierarchical and complex designs, identification of the appropriate level for tests and full reporting of outcomes
☒	☐	Estimates of effect sizes (e.g. Cohen's *d*, Pearson's *r*), indicating how they were calculated

Our web collection on statistics for biologists contains articles on many of the points above.

Software and code

Policy information about availability of computer code

Data collection	Electrophysiological and functional imaging data were collected using Matlab 2013b (with ScanImage 3.8) and Python 2.7.15. Confocal images were acquired using the Leica Application Suite X. Connectomics data were obtained from the publicly available neuprint data collection (https://neuprint.janelia.org/). hemibrain version 1.0.1 was analyzed.
Data analysis	Data were analyzed with custom-written code in Python 2.7.15 and Python 3.8.8 using scipy 1.6.2, statsmodels 0.12.2, scikit_posthocs 0.6.7 and astropy 2.0.6 packages. Confocal images were processed in Fiji/Image J 1.52e. Custom-written code is available through the G-Node GIN Data repository at https://gin.g-node.org/gammer/Ammer_et_al_2023.git

For manuscripts utilizing custom algorithms or software that are central to the research but not yet described in published literature, software must be made available to editors and reviewers. We strongly encourage code deposition in a community repository (e.g. GitHub). See the Nature Portfolio guidelines for submitting code & software for further information.

Data

Policy information about availability of data

All manuscripts must include a data availability statement. This statement should provide the following information, where applicable:

- Accession codes, unique identifiers, or web links for publicly available datasets
- A description of any restrictions on data availability
- For clinical datasets or third party data, please ensure that the statement adheres to our policy

Data that was generated in this study will be made available through the G-Node GIN Data repository at https://gin.g-node.org/gammer/Ammer_et_al_2023.git. Connectomics data is available at: https://neuprint.janelia.org/

Research involving human participants, their data, or biological material

Policy information about studies with human participants or human data. See also policy information about sex, gender (identity/presentation), and sexual orientation and race, ethnicity and racism.

Reporting on sex and gender	N/A
Reporting on race, ethnicity, or other socially relevant groupings	N/A
Population characteristics	N/A
Recruitment	N/A
Ethics oversight	N/A

Note that full information on the approval of the study protocol must also be provided in the manuscript.

Field-specific reporting

Please select the one below that is the best fit for your research. If you are not sure, read the appropriate sections before making your selection.

☒ Life sciences ☐ Behavioural & social sciences ☐ Ecological, evolutionary & environmental sciences

For a reference copy of the document with all sections, see nature.com/documents/nr-reporting-summary-flat.pdf

Life sciences study design

All studies must disclose on these points even when the disclosure is negative.

Sample size	No sample size calculations were performed before experiments. Sample sizes were chosen according to standard sample sizes in the field (e.g., Seelig & Jayaraman, Nature, 2015; Lyu et al., Nature, 2021).
Data exclusions	Data from flies that did not show any detectable calcium or voltage responses to visual stimuli were excluded.
Replication	We do not report any findings that could not be replicated or reproduced. All attempts at replication were successful.
Randomization	Experimental animals were randomly chosen from vials in which they were group-housed and were allocated to experimental groups based on their genotype. All visual stimuli were presented in a randomized manner.
Blinding	Experimenters were not blinded to genotypes, which would be impractical. However data collection and analysis was automated and equivalent for different genotypes.

Reporting for specific materials, systems and methods

We require information from authors about some types of materials, experimental systems and methods used in many studies. Here, indicate whether each material, system or method listed is relevant to your study. If you are not sure if a list item applies to your research, read the appropriate section before selecting a response.

Materials & experimental systems

n/a	Involved in the study
☐	☒ Antibodies
☒	☐ Eukaryotic cell lines
☒	☐ Palaeontology and archaeology
☐	☒ Animals and other organisms
☒	☐ Clinical data
☒	☐ Dual use research of concern
☒	☐ Plants

Methods

n/a	Involved in the study
☒	☐ ChIP-seq
☒	☐ Flow cytometry
☒	☐ MRI-based neuroimaging

Antibodies

Antibodies used	anti-brp (nc82) mouse monoclonal antibody (DSHB) RRID: AB_2314866, myeloma strain: P3X63Ag8.653; anti-GFP chicken polyclonal antibody (Rockland) Cat# 600-901-215S; anti-DsRed rabbit polyclonal antibody (Takara Bio) RRID: AB_10013483; donkey anti-chicken-Alexa-488 (Jackson Immuno Research) Cat# 703-545-155; goat anti-rabbit-Alexa-568 (Invitrogen) Cat# A-11011; goat anti-mouse-Alexa-647 (Invitrogen) Cat# A32728
Validation	All primary antibodies used in this study are widely used in the field of Drosophila neurobiology. Information on validation is available at the manufacturers website at: https://dshb.biology.uiowa.edu/nc82 for anti-brp; https://www.rockland.com/categories/primary-antibodies/gfp-antibody-600-901-215/ for anti-GFP and https://www.takarabio.com/documents/Certificate%20of%20Analysis/632496/632496-101717.pdf for anti-DsRed.

Animals and other research organisms

Policy information about studies involving animals; ARRIVE guidelines recommended for reporting animal research, and Sex and Gender in Research

Laboratory animals	Flies of the species Drosophila melanogaster were used for all experiments. Age ranged between 1 and 7 days.
Wild animals	No wild animals were used in this study.
Reporting on sex	Due to their larger body size, only female flies were used in this study.
Field-collected samples	No field-collected samples were used in this study.
Ethics oversight	No ethical approval is required for research on Drosophila melanogaster in Germany.

Note that full information on the approval of the study protocol must also be provided in the manuscript.
