# Supplementary material for: Multilevel visual motion opponency in Drosophila
Source: Nat Neurosci. 2023 Oct 2;26(11):1894–905. doi: 10.1038/s41593-023-01443-z (PMC10620086; doi:10.1038/s41593-023-01443-z)
Supplement: Supplementary file 5 — Source data. [file 41593_2023_1443_MOESM5_ESM.zip › Figure 1/Optogenetic_T4T5_oscillations/Laboratory_notebook_scan.pdf]

7/12 (Wed)

norpA[7] ; -H134R ; T4T5-Gal4[x114]

♀ collected Tue 14.00, kept on [1mM ATR/yeast] until experiment Wed 16.00
prepared Mon p.M.

→ fill VS1-3?

Prep 1 cell 1: VS cell -39mV @ 8MΩ

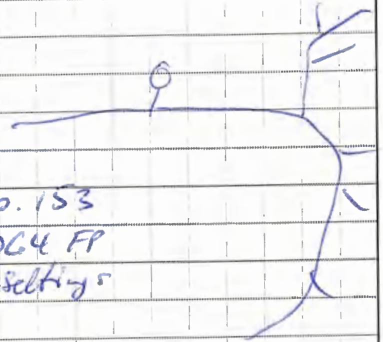

	16.41	current/flicker					
1	16.43	8x	1000ms	blue light	FP 9		
	16.44	full DT					
x 2	.46	8x	2ms	blue light	FP 9		
3	.47	8x	1000ms	blue	FP 9		
~~4~~	~~.49~~	~~8x~~	~~1000ms~~	~~blue~~			mW/mm²
x 5	.50	8x	2ms	blue	FP 9		
x 6	.51	— " —		"	FP (14)!	]	5.4
x 7	.52	— " —		"	FP (14)!	]	
x 8	.53	— " —		green	FP (13)	]	
x 9	.54	— " —		green	FP (13)	]	
	.56	current/flicker					
10	.57	8x	2ms	blue	FP 7	]	0.4
11	.58	— " —			FP 7	]	
12	17.01	— " —			FP 9	]	1.7
13	.02	— " —			FP 9	]	
14	17.03	— " —			FP 1	]	16
15	.04	— " —			FP 1	]	
	.05	current/flicker					
	.06	— " —					
16	.07	8x	1000ms	blue	FP 10		
17	.08	— " —					
18	.09	8x	1000ms	green	FP 13		
19	.10	— " —					
20	.11	— " —					

→ see p. 153 for 0.64 FP settings

27	17.20	4x 1000ms blue no current
28	.21	— " — cell hyperpolarised -50mV (added to Vrest!)
29	.22	-80mV (added to Vrest)
30	.23	no current
31	.23	+50mV
32	.24	+100mV

		ISI	
21	17.13	5	20x 2ms blue light FP 10
22	.14	10	
23	.14	15	
24	.15	20	
25	.15	25	
26	.17	3	

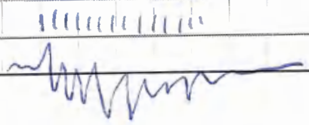

AB - 60 - 170
BOOK PAGE

Same as previous page!

norpA^7 ; H134R ; T4T5-Gal4^[illegible]

ATR ~1d

7/12 (Wed)

Prep2 cell1 — no [illegible] VS/HS — -36mV — e.8MΩ

18.23 current/flicker

1 18.24 4x 1000ms blue FP9
2 .24 '' ''

.25] current/flicker
.27]

3 18.28 8x 2ms blue FP9 — see p. 153 for ND4 settings
4 .29 '' '' FP10
5 .30 '' '' FP14
6 .32 '' '' FP 1

.33 current/flicker

Good!
Prep2 cell2 — VS cell! → fill VS1-3?

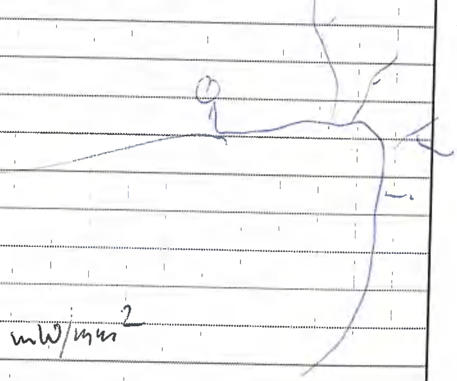

press.on / see cham[illegible] / [illegible] in LP. / OK ✓

18.48] current/flicker
.48]

1 18.49 8x 1000ms blue FP10

mW/mm²

2	18.50	8x	2ms	blue	FP10	]	2.8
3	.51	''		''		]	
4	.52	8x	2ms	blue	FP14	]	5.4
5	.53	''		''		]	
6	.54	8x	2ms	blue	FP8	]	0.9
7	.55	''		''		]	
8	.57	8x	2ms	blue	FP9	]	1.7
9	.58	''		''		]	
10	.59	8x	2ms	blue	FP10	]	2.8
11	19.00	''		''		]	
12	19.01	8x	1000ms	blue	FP10		ca. 2.8 mW/mm²
13	19.02	8x	1000ms	green	FP13		ca. 2.2 mW/mm²
14	19.04	8x	1000ms	blue	FP 9		ca. 1.7 mW/mm²
15	.06	same but +50mV injected					
16	.07	same but -50mV ''					
17	.08	'' '' +100mV ''					
