# Supplementary figures and images for: Multilevel visual motion opponency in Drosophila
Source: Nat Neurosci. 2023 Oct 2;26(11):1894–905. doi: 10.1038/s41593-023-01443-z (PMC10620086; doi:10.1038/s41593-023-01443-z)

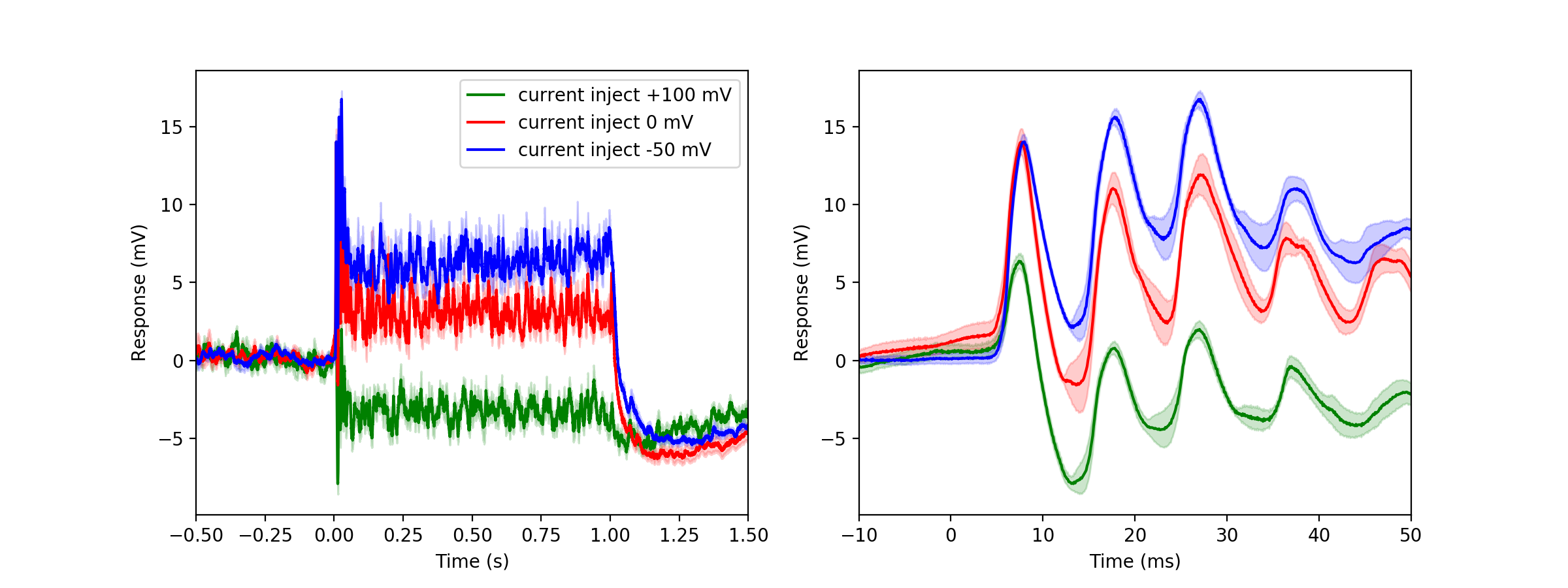

Supplement: Supplementary file 5 — Source data. [file 41593_2023_1443_MOESM5_ESM.zip › Figure 1/Optogenetic_T4T5_oscillations/Figure_T4T5_stim_VS_response.png]

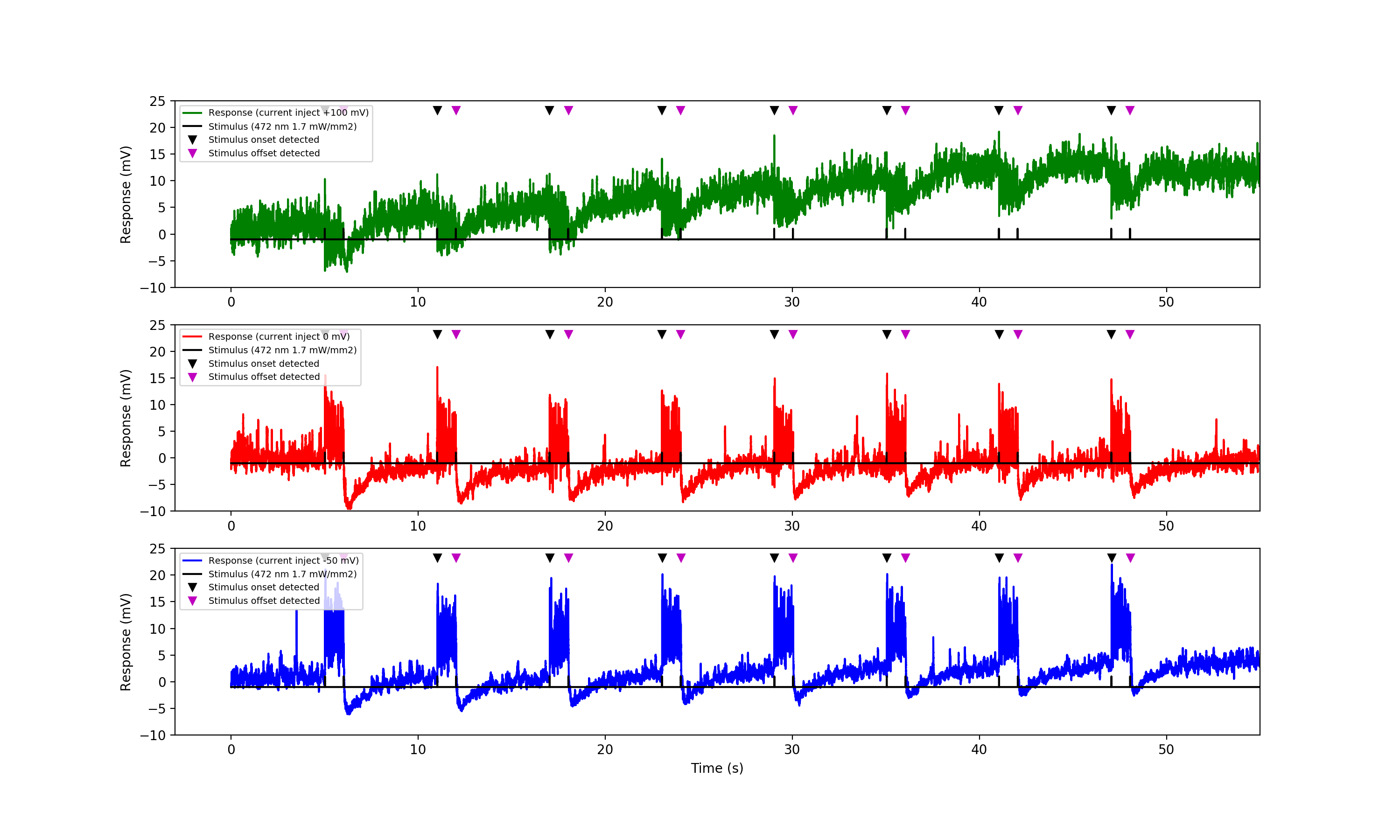

Supplement: Supplementary file 5 — Source data. [file 41593_2023_1443_MOESM5_ESM.zip › Figure 1/Optogenetic_T4T5_oscillations/Figure_T4T5_stim_VS_response_trials.png]

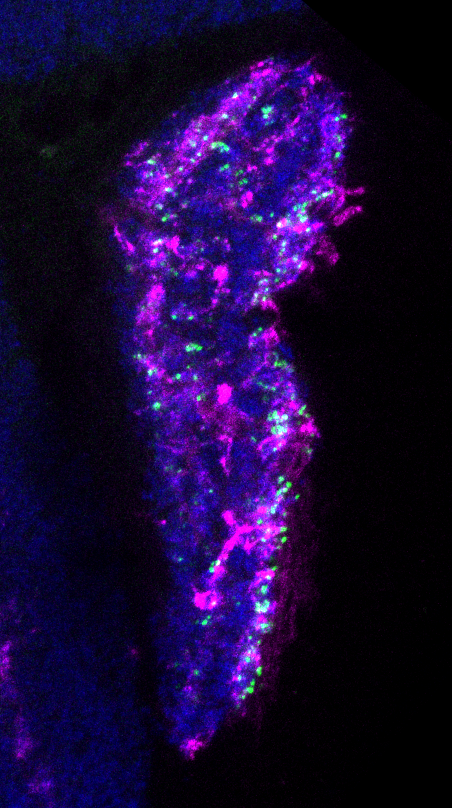

Supplement: Supplementary file 8 — Source data and image files. [file 41593_2023_1443_MOESM8_ESM.zip › Figure 4/1a_LPTCs_UAS-GluCl.png]

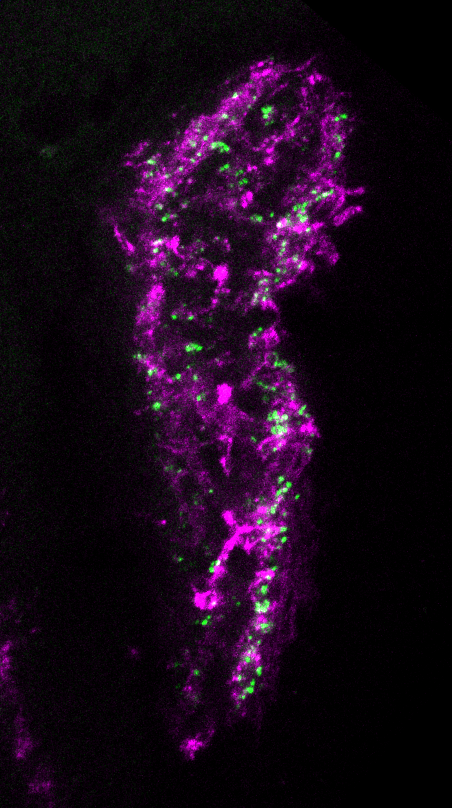

Supplement: Supplementary file 8 — Source data and image files. [file 41593_2023_1443_MOESM8_ESM.zip › Figure 4/1a_LPTCs_UAS-GluCl_wo_nc82.png]

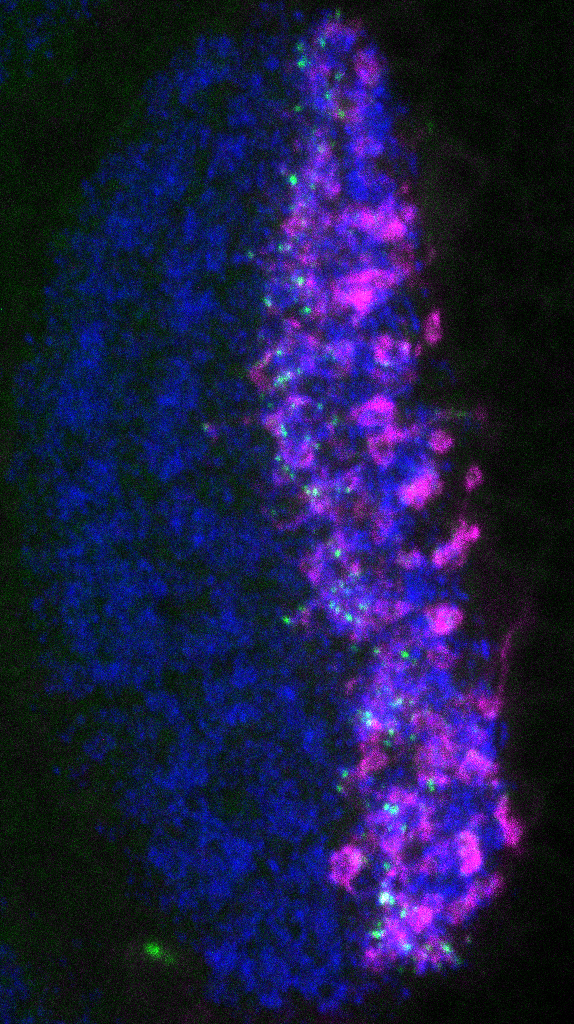

Supplement: Supplementary file 8 — Source data and image files. [file 41593_2023_1443_MOESM8_ESM.zip › Figure 4/1b_LPi3-4_UAS-GluCl.png]

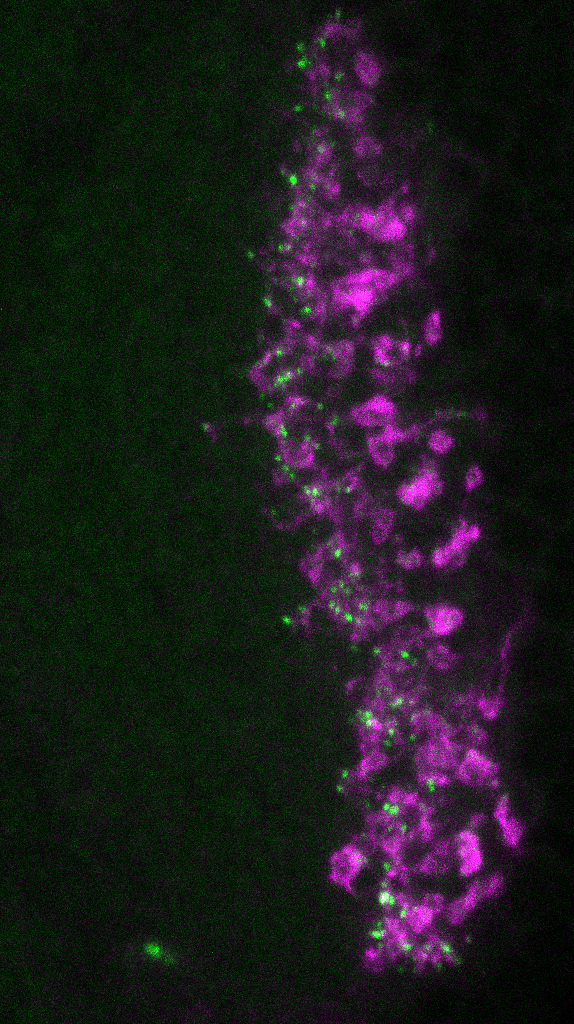

Supplement: Supplementary file 8 — Source data and image files. [file 41593_2023_1443_MOESM8_ESM.zip › Figure 4/1b_LPi3-4_UAS-GluCl_wo_nc82.png]

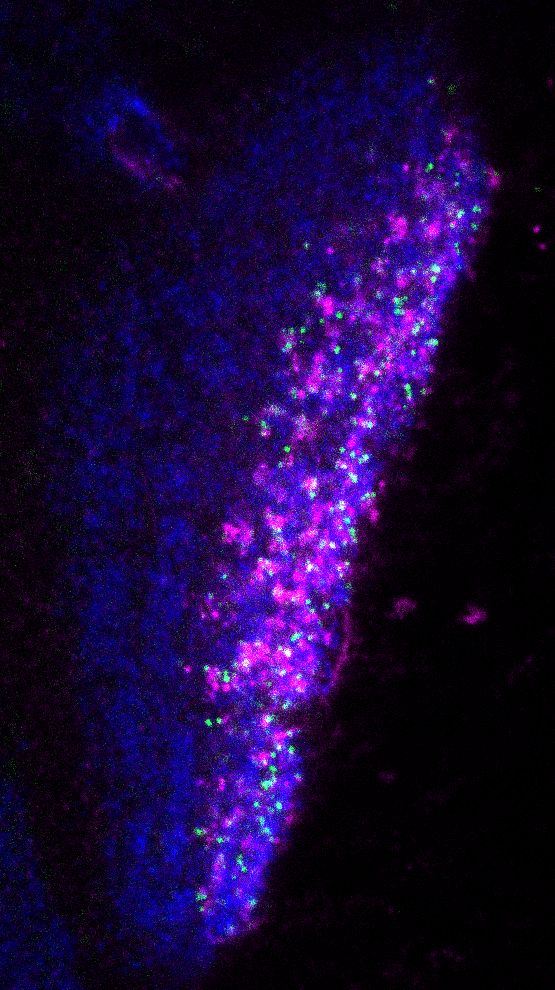

Supplement: Supplementary file 8 — Source data and image files. [file 41593_2023_1443_MOESM8_ESM.zip › Figure 4/1c_LPi4-3_UAS-GluCl.png]

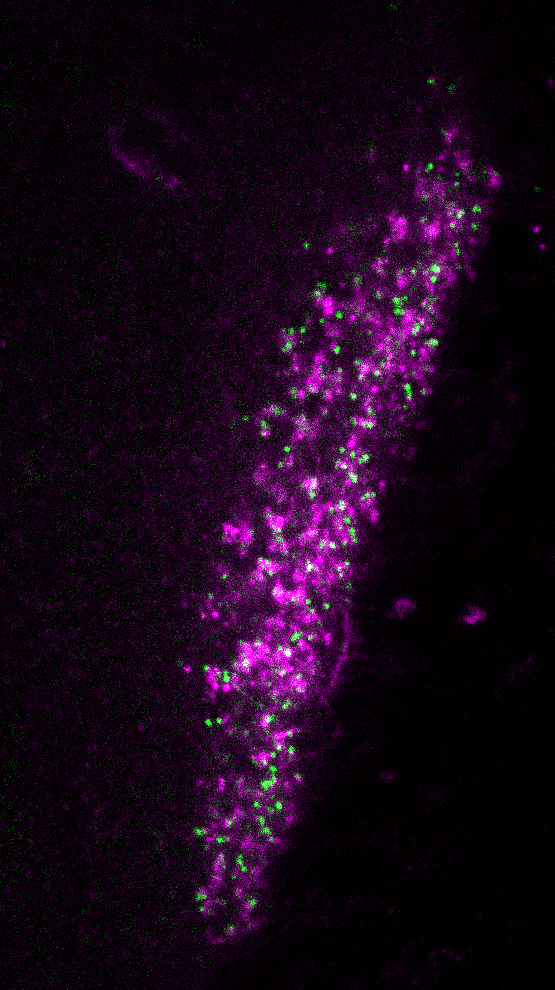

Supplement: Supplementary file 8 — Source data and image files. [file 41593_2023_1443_MOESM8_ESM.zip › Figure 4/1c_LPi4-3_UAS-GluCl_wo_nc82.png]

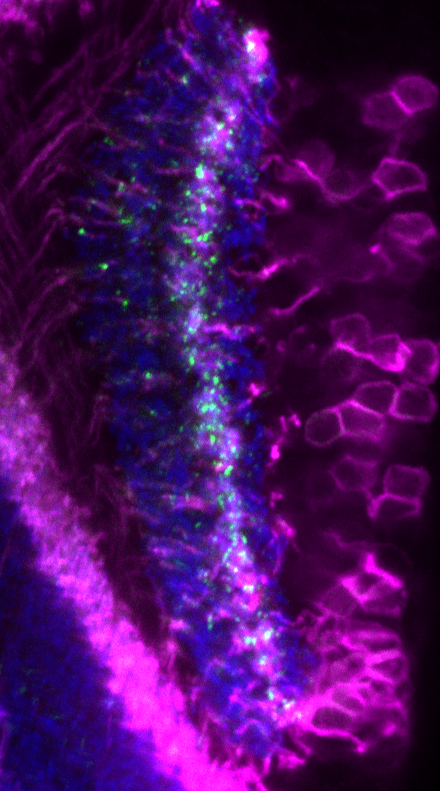

Supplement: Supplementary file 8 — Source data and image files. [file 41593_2023_1443_MOESM8_ESM.zip › Figure 4/1d_T4T5c_UAS-GluCl.png]

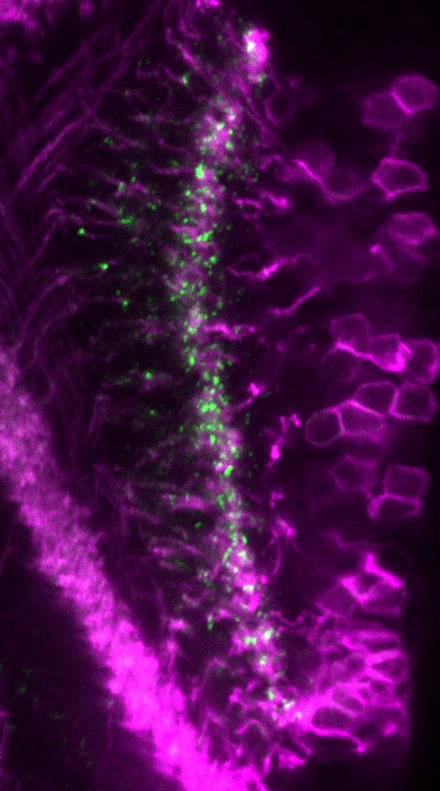

Supplement: Supplementary file 8 — Source data and image files. [file 41593_2023_1443_MOESM8_ESM.zip › Figure 4/1d_T4T5c_UAS-GluCl_wo_nc82.png]

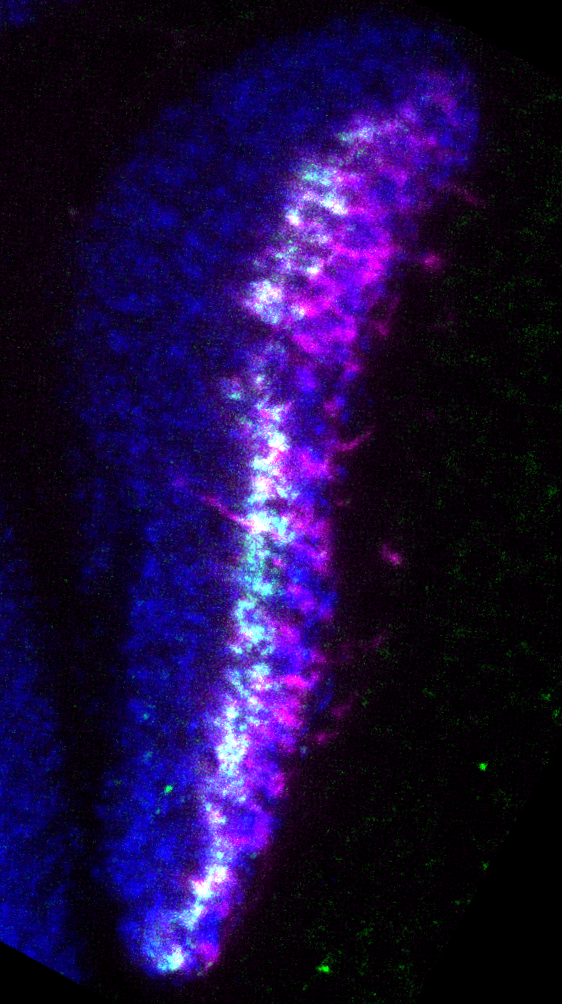

Supplement: Supplementary file 15 — Image files. [file 41593_2023_1443_MOESM15_ESM.zip › Figure S4/S4a_LPi3-4_UAS-Da7.png]

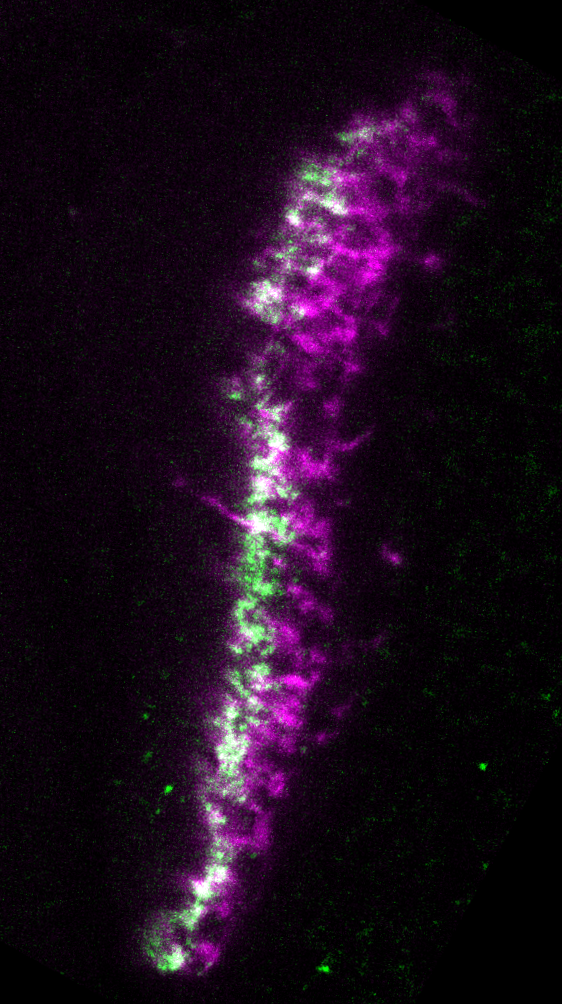

Supplement: Supplementary file 15 — Image files. [file 41593_2023_1443_MOESM15_ESM.zip › Figure S4/S4a_LPi3-4_UAS-Da7_wo_nc82.png]

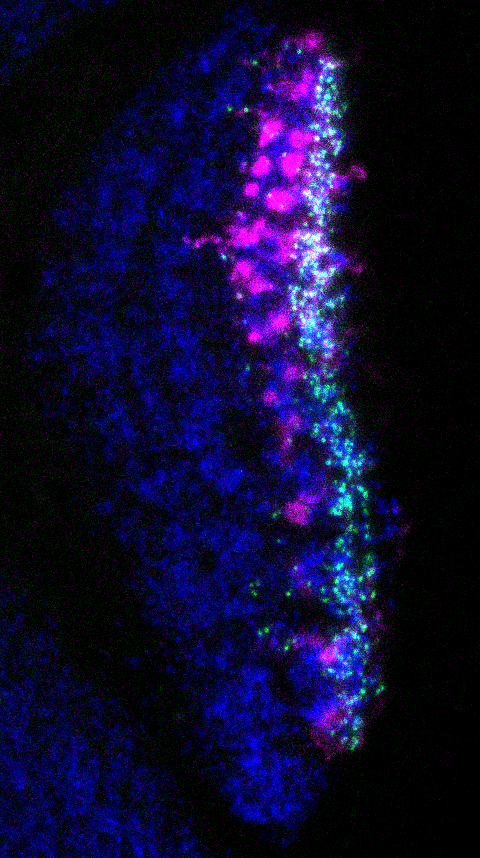

Supplement: Supplementary file 15 — Image files. [file 41593_2023_1443_MOESM15_ESM.zip › Figure S4/S4b_LPi4-3_UAS-Da7.png]

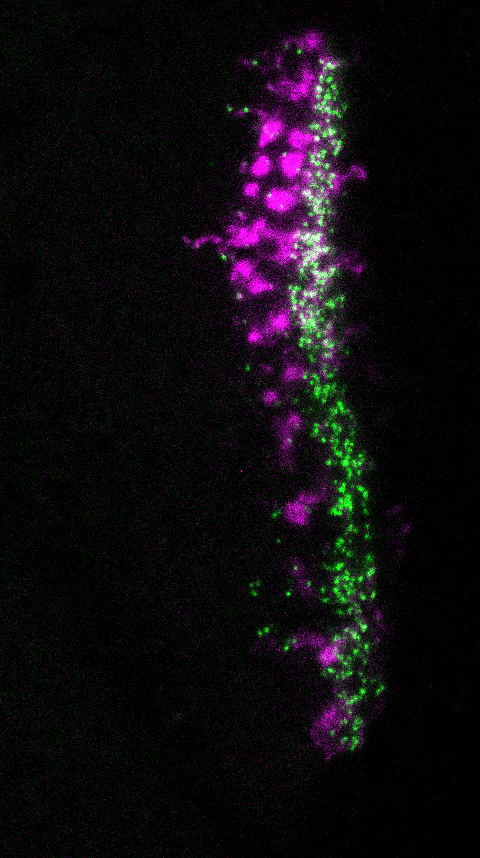

Supplement: Supplementary file 15 — Image files. [file 41593_2023_1443_MOESM15_ESM.zip › Figure S4/S4b_LPi4-3_UAS-Da7_wo_nc82.png]
